# Detection of Pancreatic Ductal Adenocarcinoma by A qPCR-based Normalizer-free Circulating Extracellular Vesicles RNA Signature

**DOI:** 10.7150/jca.50716

**Published:** 2021-01-01

**Authors:** Da Qin, Yu Zhao, Qingdong Guo, Shengtao Zhu, Shutian Zhang, Li Min

**Affiliations:** 1 Department of Gastroenterology, Beijing Friendship Hospital, Capital Medical University, National Clinical Research Center for Digestive Disease, Beijing Digestive Disease Center, Beijing Key Laboratory for Precancerous Lesion of Digestive Disease, Beijing, 100050, P. R. China.

**Keywords:** PDAC, EVs-derived RNAs, circulation biomarker, standardization, RT-qPCR

## Abstract

**Background:** Pancreatic ductal adenocarcinoma (PDAC) is difficult to diagnose and many efforts have been made to evaluate EVs-derived RNAs as biomarkers to predict PDAC. However, lack of robust internal references largely limited their clinical application. Here we proposed an RNA ratio-based, normalizer-free algorithm to quantitate EVs-derived RNAs in PDAC.

**Methods:** Differentially expressed RNAs in the training group were identified using “limma” package. The ratio of any two candidate RNAs in the same sample was calculated and used as a new biomarker. LASSO regression was performed to build prediction models based on those RNA ratios. RNA-seq data of 116 plasma samples and RT-qPCR data of 111 plasma samples were used for internal and external validation, separately. Three algorithms (lasso regression, logistic regression, and SVM) were compared to improve the performance of this RNA signature.

**Results:** We developed an RNA-ratio based prediction model which comprised eight EVs-derived RNAs, including FBXO7, MORF4L1, DDX17, TALDO1, AHNAK, TUBA1B, CD44, and SETD3. This model could well differentiate PDAC patients with a minimal AUC of 0.86 in internal verification using testing group. External validation using RT-qPCR data also exhibited a good classifier ability with an AUC of 0.89 when distinguishing PDAC from healthy controls.

**Conclusion:** We've developed a qPCR-based, normalizer-free circulating EVs RNA classifier, which could well distinguish PDAC patients from noncancerous controls.

## Introduction

Pancreatic cancer (PC) is predicted to be the second most common cause of cancer-related death around the world by 2030^1^. Pancreatic ductal adenocarcinoma (PDAC) is the most common subtype of PC. More than 85% of PC confirmed to be PDAC after histological confirmation^2^. PDAC has properties of early metastatic potential and resistance to existing treatment such as chemotherapy or radiotherapy, which leads to its poor prognosis and high mortality. The 5-year survival rate of PDAC is less than 8%^3^,^4^. Thus, screening tools which could identify PDAC from general population in its resectable stage are crucial and urgently needed. Traditional biomarkers such as CA-199 and CEA have poor sensitivity and specificity, resulting in misdiagnosis. Emerging biomarkers such as plasma miRNAs, plasma lncRNAs, circulation tumor DNAs, and plasma extracellular vesicles (EVs) exhibited better accuracy in detecting various types of cancer^4^,^5^.

EVs are membrane-encapsulated heterogenous vesicles delivering various macromolecules including proteins, miRNAs, lncRNAs, mRNAs, or lipids between different microenvironments, resulting in initiation of various biological processes due to their different cargoes^6^. Tumor cells produce a large number of EVs, and EVs cargoes play important roles in tumorigenesis, angiogenesis, metastasis, and other pathological processes. Tumor-specific expression patterns and nonnegligible biological functions make EVs one of the excellent biomarker sources. Our previous study has also proved that plasma EVs-derived RNAs provided higher classifier ability than RNAs directly isolated from plasma and are more suitable to be biomarkers^7^.

There are several methods could be used to detect EVs-derived RNAs such as next generation sequencing (NGS), RNA chip, and RT-qPCR. NGS and RNA chip are relatively accurate but not suitable for large-scale clinical application because of their high-cost. Real-time quantitative PCR (RT-qPCR) is an alternative way to detect RNAs which is more cost-effective than omics tools. However, the absence of a well-established normalizer for plasma EVs derived RNA quantification largely hampered the development of qPCR-based assays, and resulted in unfavorable data consistency and reproducibility across different studies^8^-^13^.

To solve this problem, here we proposed a ratio-based normalization for circulating EVs derived long RNA data. The ratio of any two candidate RNAs in the same sample was calculated and used as a new biomarker, and then the ratios were compared between different groups instead of a single RNA level. Considering the two RNAs in an RNA-pair are simultaneously measured under the same condition, their ratio could reflect the true difference among samples by canceling systematic biases. So instead of absolute quantification of a single RNA, we established a new scoring system based on RNA-pairs, which enables us to build a quantitative RNA classifier without an RNA internal reference.

## Materials and Methods

### Patients Cohorts

**Figure 1:**
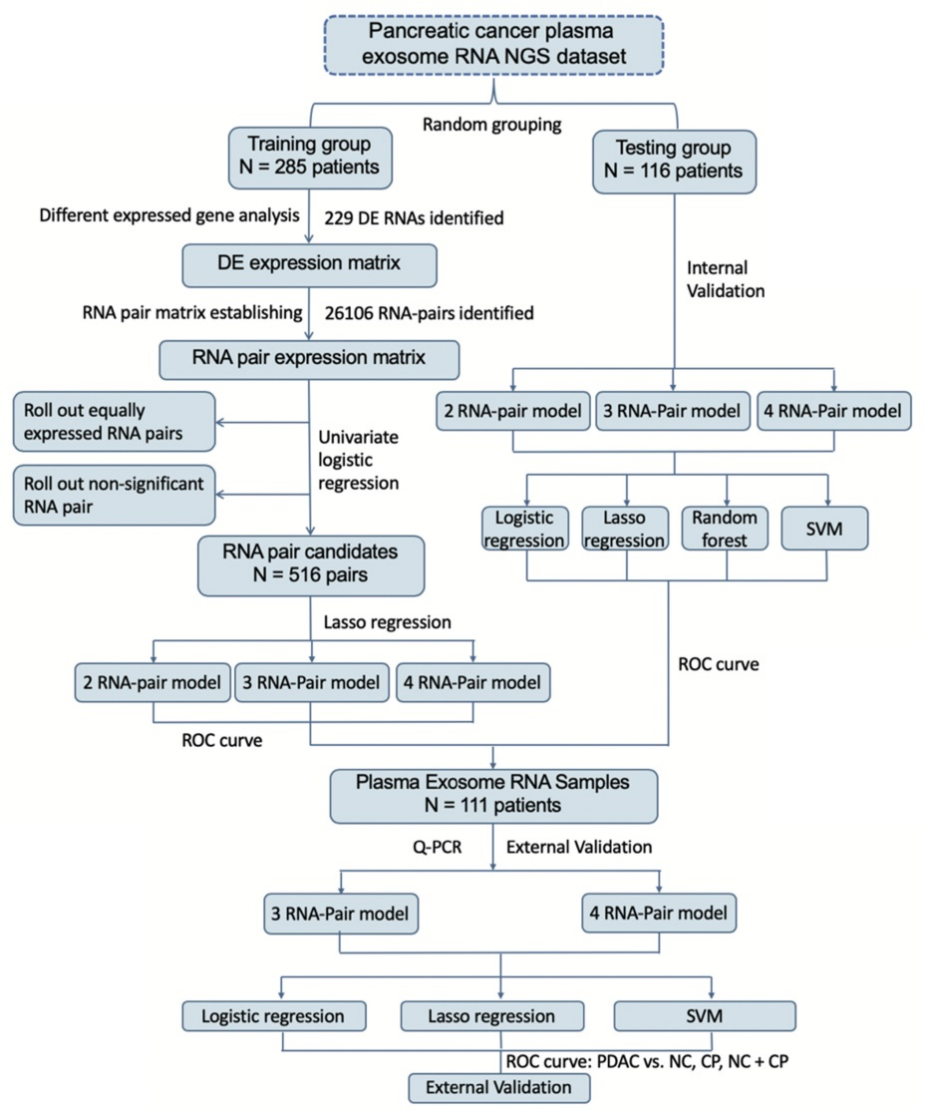
** Flowchart of study.** Establishment of a new scoring system based on RNA-pairs. Briefly, the GSE133684 dataset was randomly divided into a training group and a testing group. DEGs were identified and constructed RNA-pairs. PDAC prediction models were built based on 2-4 RNA-pairs, and verified by both internal NGS data and external RT-qPCR data.

Circulating EVs-derived long RNA NGS data from GSE133684^14^ in GEO database were obtained for analysis. Clinical details of GSE133684 were shown in Table 1. GSE133684 was randomly divided into a training group and a testing group. Training group was used to establish RNA pair prediction model and testing group was used for internal validation. 111 receivers including PDAC patients, chronic pancreatitis patients (CP) and normal controls (NC) from Beijing Friendship Hospital were recruited for external validation of these prediction models. Inclusion criteria of patients in external validation group were (1) patients pathologically diagnosed PDAC or CP in Beijing Friendship Hospital during 2018-1-1 to 2020-1-1; (2) patients agreed to attend this research and assigned the informed consent forms; (3) blood samples were available before surgery or treatment. Exclusion criteria were (1) other kinds of tumors were diagnosed in the same patient; (2) patients with IgG4-related disease or any other autoimmune disease were not proper to attend this study; (3) patients with severe inflammation or septic shock were not able to attend this study; (4) patients with severe coagulation dysfunction; (5) patients refused to attend this research. Clinical details of these subjects were also shown in Table 1. All participants had signed the informed consent, and this study was approved by the ethics committee of Beijing Friendship Hospital. Flowchart of this study was shown in Figure 1.

### Differentially expressed RNA analysis and GO analysis

For NGS data in training group, RNAs which TPM lower than 50 were excluded because of the difficulty in qPCR verification. Differentially expressed (DE) RNA analysis was performed to training group using “limma” package in R 3.5.2^15^. To shrink rang of DE RNAs, cut-off levels of DE RNAs were identified as |Log fold change| > 3 and adjusted p-value < 0.0001. Volcano plot and heatmap were also performed to show the expression differences between PDAC patients and healthy controls. Moreover, to better understand the biological processes (BP), cellular components (CC) and molecular features (MF) of these DE RNAs, Gene Ontology (GO) analysis was also performed by using “clusterProfiler” package in R^16^.

### RNA-pair matrix construction and prediction model construction

RNA pair ratio was calculated from any two DE RNAs expression values in the same sample. Univariate logistic regression was subsequently performed for each RNA pair to roll out pairs not significantly associated with PDAC occurrence and constructed an RNA pair matrix of training group. Next, we used lasso regression to select variables from RNA pair matrix under 7-fold cross-validation by “glmnet” package in R. Prediction models enrolled 2, 3, 4 RNA pairs were established by a step-wise variable selection process by controlling lambda values in lasso regression. Then, logistic prediction models, support vector machine (SVM) models and Lasso regression models for each of these RNA pair signatures were constructed by “glm” function, “e1071” package and “glmnet” package^17^ in R, separately. Receiver operator characteristic (ROC) curves and area under the curve (AUC) of all these prediction models were calculated to evaluate their performance.

### Internal verification by NGS data in testing group

Testing group of GSE133684 was used for internal verification of these models. ROC curves of these models were drawn by using “plotROC” package^18^ and “ggplot2” package in R. AUC values of internal verification for each model were also calculated.

### EVs isolation

This study isolated EVs of plasma samples of 111 PDAC patients, CP patients and normal controls. 3 mL plasma sample was collected from each subject. We performed ultracentrifugation (UC) method for EVs isolation. Firstly, we centrifugated plasma samples at 3000 × g for 15min after thawing at 37°C in order to remove cell debris. Then, we diluted supernatant of each sample by 7-fold volume of phosphate-buffered saline (PBS). Next we centrifuged the mix again at 13,000 × g for 30min and a 0.22μm filter was applied in order to filter large vesicles. Then, a P50AT2-986 rotor (CP100NX; Hitachi, Brea, CA, USA) was used at 150,000 × g, 4°C for 4h to pellet EVs. Pellet was resuspended in PBS and centrifuged again at 150,000 × g 4°C for 2h. Finally, the EVs enriched fraction pellet was re-suspended in 100µL PBS.

### EV protein quantification

Pierce BCA Protein Assay Kit (Thermo Scientific, Product No. 23,225) were used to quantify the protein concentration of EVs-enriched fractions following the manufacturer's protocol. 10μL of each standard and samples were pipetted into 96-Well Plates. After mixture of 200μL of the WR to each well and shaking for 30 second on the shaker, plates were covered and incubated at 37°C for 30min. Then we measured their absorbance at 562nm on the plate reader. Finally, we build a standard curve and quantified EVs-enriched fraction samples.

### Nanoparticle tracking analysis (NTA)

The ZetaView PMX 110 (Particle Metrix, Meerbusch, Germany) with 405 nm laser was applied to find out size and quantity of EVs in concentrations ranged 1x10^7^ /mL to 1x10^9^ /mL. We took a 60-second video with a frame rate of 30 frames/sec, and analyzed it by using NTA software (ZetaView 8.02.28).

### Transmission electron microscopy (TEM)

We next placed 20µL EVs enriched solution on a copper mesh for 10min in room temperature. Then, we contrasted solution with uranyloxalate solution for 1min after washed by sterile distilled water. The solution was subsequently dried for 2min under incandescent light. The copper mesh was then photographed under a TEM (JEOL-JEM1400, Tokyo, Japan).

### Western blot

Special markers for extracellular vesicles have been previously reported. In the present study, we used a combination of two positive markers (CD63 and TSG101) and one negative markers (calnexin) to characterize the exosomes we extracted. Rabbit polyclonal antibody CD63 (sc-5275, Santa Cruz, CA, USA), TSG101 (sc-13611, Santa Cruz, CA, USA) and calnexin (10427-2-AP, Promega, Madison, WI) were used in Western Blot procedures. The protein bands were detected using an enhanced chemiluminescence system (Bio-Rad, USA).

### RNA isolation and RT-qPCR validation

Total RNA isolated was extracted and purified from plasma EVs using the miRNeasy Mini kit following the manufacturer's protocol (No. 217004, Qiagen, Hilden, Germany). A total RNA was reverse-transcribed and cDNA was synthesized using PrimeScript^TM^ RT Master Mix (TAKARA RR036a). The amplification of cDNA was performed in 10-μL reaction system following the SYBRGREEN life assays manufacturer's instructions. Primer sequence was shown in Table S1. Expression ratios of any two of these RNAs were calculated separately and taken into prediction models. ROC curves were drawn by using “plotROC” package and “ggplot2” package. AUC values of external verification by RT-qPCR were also calculated.

### Statistical analysis

All statistical analyses were calculated by R software (version 3.5.2; https://www.r-project.org/). DE RNAs were identified by “limma” package (version 3.38.3). ROC curves were graphed using “plotROC” package (version 2.2.1). Lasso regression was performed using “glmnet” package (version 2.0-18). SVM were performed using e1071 package (version 1.7-2). All statistical significance was defined as P < 0.05.

## Results

### Differentially expressed RNA identification

**Figure 2:**
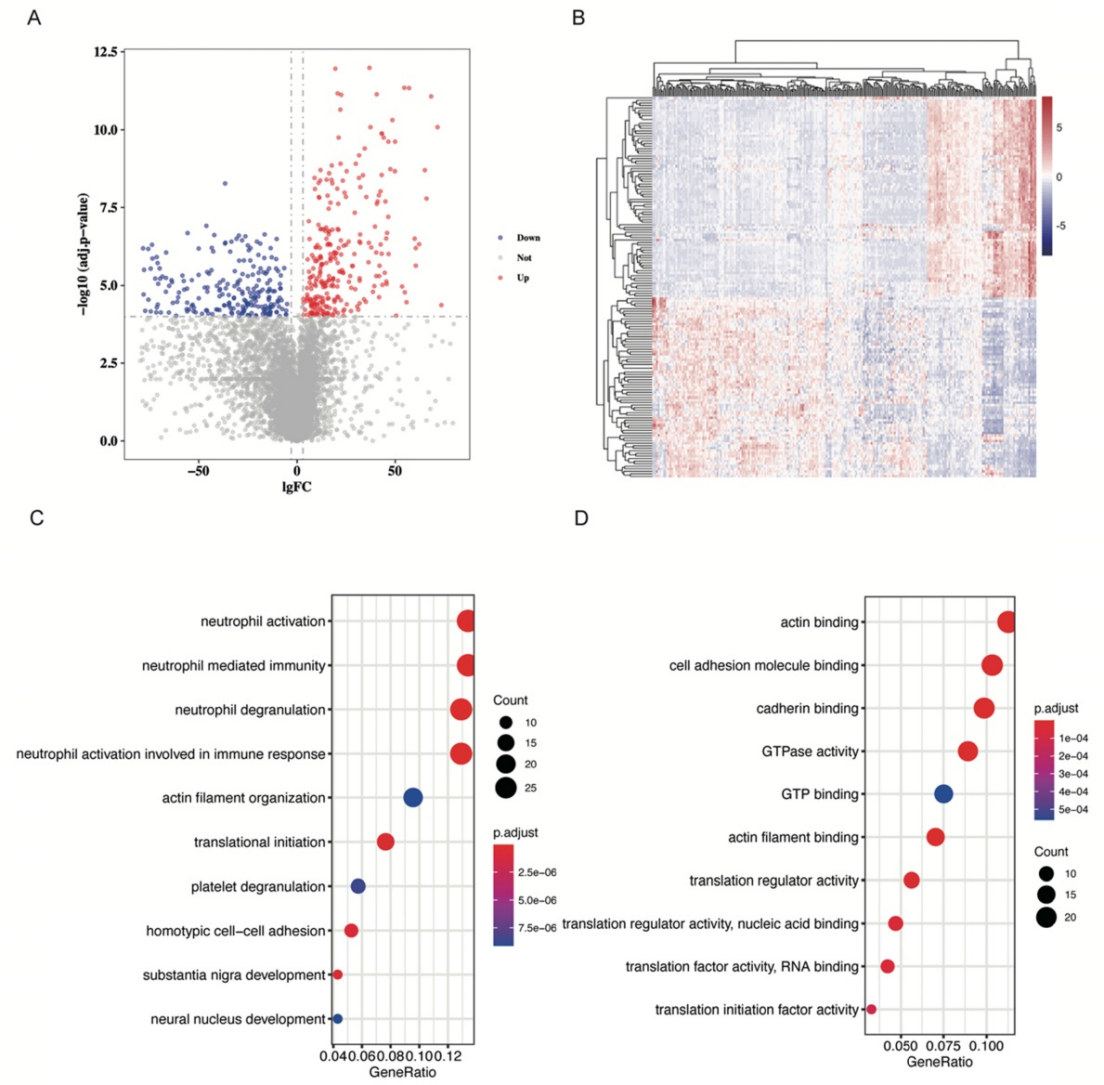
** DE RNAs identification and GO analysis. A.** Volcano plot showing DE RNAs. DE RNAs were identified as |Log fold change| > 3 and adjusted p-value < 0.0001. Red dots represent up-regulated RNAs, and blue dots represent down-regulated RNAs.** B.** Heatmap of DE RNAs.** C.** Dot plot to show DE RNAs enriched biological processes. Neutrophil activation and neutrophil mediated immunity were significantly enriched.** D.** Dot plot to show DE RNAs enriched molecular function. Actin binding and cell adhesion molecule binding were significantly enriched.

Samples in the GSE133684 dataset were randomly divided into two cohorts, and 287 low-abundant RNAs with an average TPM lower than 50 were excluded for the difficulty in qPCR quantification. With cut-off levels of |Log fold change| > 3 and adjusted p-value < 0.0001, 229 differentially expressed RNAs were identified in the training cohort (Table S2). Volcano plot and heatmap of these DE RNAs were shown in Figure 2A and 2B, separately.

### GO analysis of differentially expressed RNAs

GO analysis was performed in these differentially expressed RNAs by “clusterProfiler” package in R. We find that DE RNAs were significantly enriched in neutrophil related biological processes (BP) such as neutrophil activation and neutrophil mediated immunity (P < 0.001, Figure 2C, Figure S1A, 1B). CD44, NCKAP1L, and DOCK2 play important roles in these biological processes (Figure S1C). As for molecular functions (MF) of RNAs, we found that they were significantly enriched in actin binding and cell adhesion molecule binding (P < 0.001, Figure 2D, Figure S1D, 1E). EEF2 and AHNAK participated in these GO terms (Figure S1F). Furthermore, we explored cellular component (CC) information of DE RNAs and found that they were mostly enriched in focal adhesion and cell-substrate junction (P < 0.001, Figure S1G, 1H). CD44 and AHNAK played crucial roles in these GO terms (Figure S1I).

### RNA-pair matrix establishment and construction of prediction models

**Figure 3:**
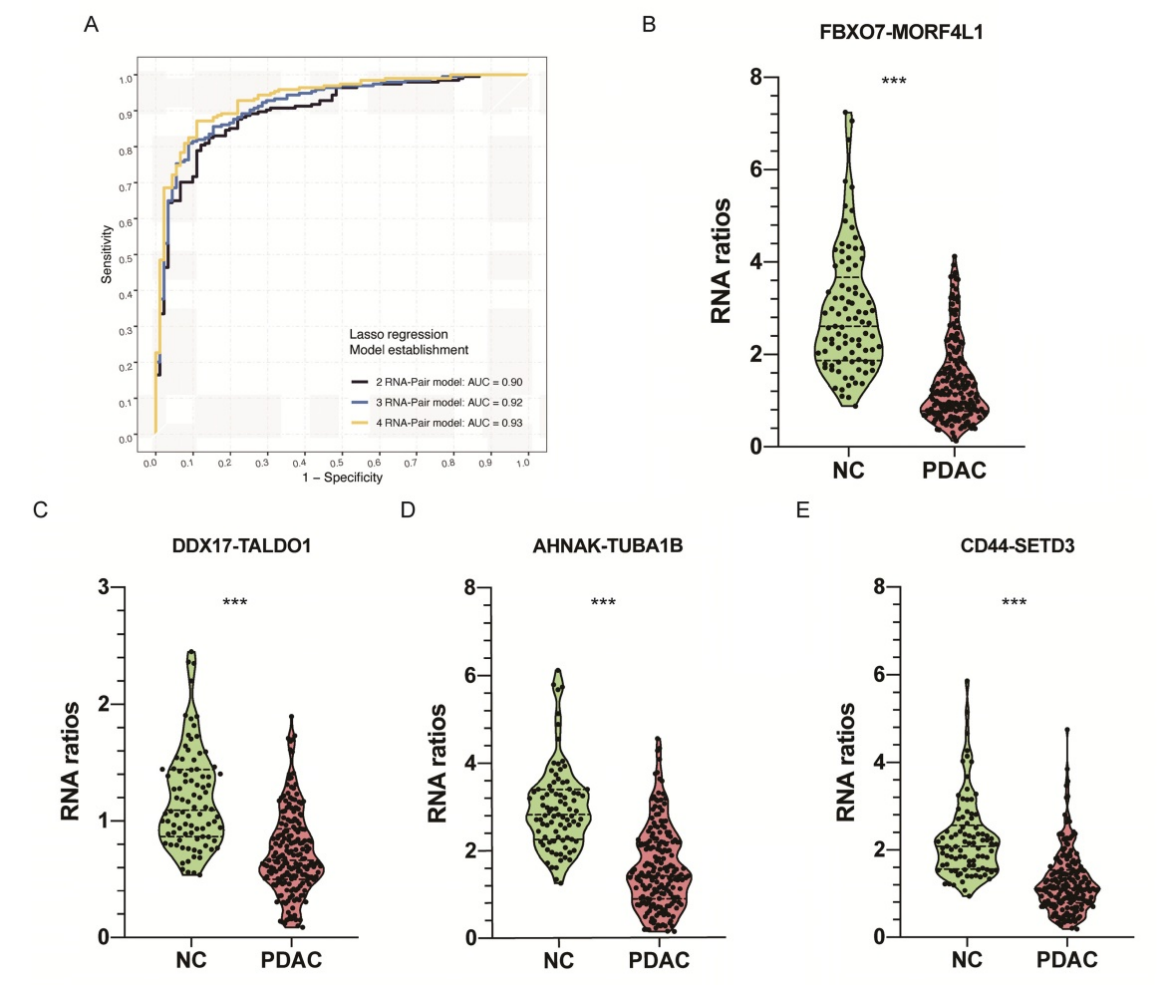
** Identification of EVs derived RNA pairs to detect PDAC. A.** Lasso regression was applied in variable selection and constructed three RNA-pair based models with 2-4 RNA-pairs by controlling lambda values in the regression.** B-E.** Violin plot showing RNA ratios distribution and differences between PDAC and NC group. ***: P < 0.001.

We paired any two of 229 candidate RNAs and conducted an RNA-pair matrix containing 26106 RNA-pairs. Among those RNA-pairs, 516 candidates achieved an AUC above 0.8 and were selected for the construction of prediction models. Lasso regression was applied in variable selection and three RNA-pair signatures were constructed by controlling lambda values in the regression (Figure 3A, Table 2, Figure S2A). Violin plot representing expression level between PDAC patients and healthy controls of these RNA-pair ratios were shown in Figure 3B-E. Ratios of these 4 RNA-pairs in healthy controls were significantly higher than those in PDAC patients (P < 0.001).

### Internal validation of prediction models in the testing cohort

**Figure 4:**
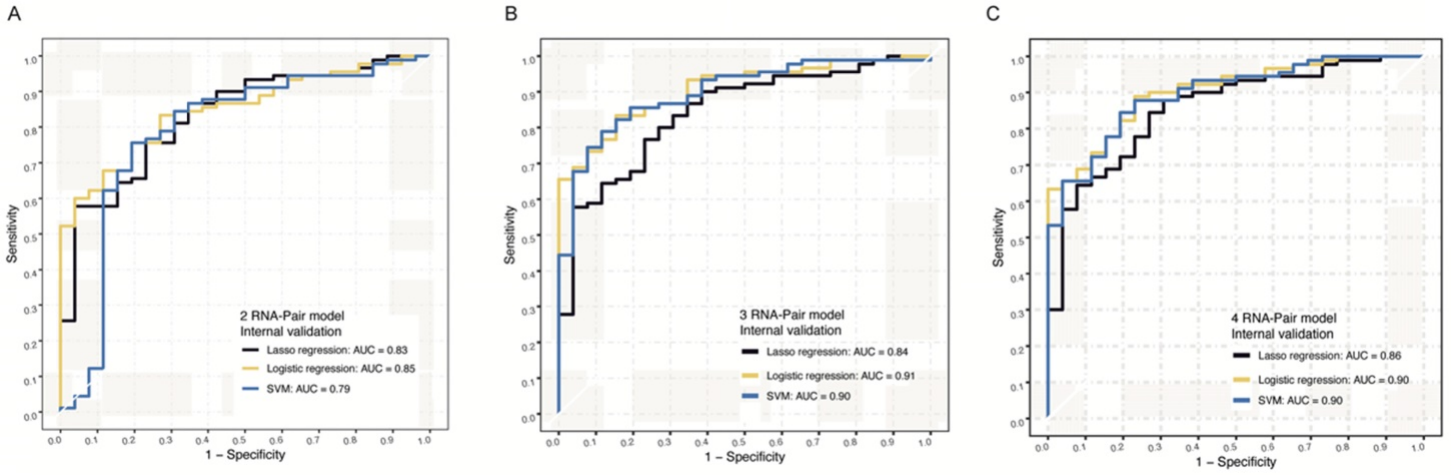
** Internal NGS verification of RNA-pair based models. A.** The ROC of models constructed by 2 RNA-pair with different algorithms in internal NGS verification.** B.** The ROC of models constructed by 3 RNA-pair with different algorithms in internal NGS verification.** C.** The ROC of models constructed by 4 RNA-pair with different algorithms in internal NGS verification.

NGS data from 116 samples were used for internal validation, and we constructed Lasso regression models, logistic models, and SVM models for internal validation of these signatures. For 2 RNA pair model, AUC values of Lasso regression algorithm, logistic algorithm, and SVM algorithm were 0.83, 0.85 and 0.79, separately (Figure 4A). For 3 RNA pair model, AUC values of these 3 algorithms were 0.84, 0.91 and 0.90 (Figure 4B), separately. For 4 RNA pair model, Logistic algorithm and algorithm model achieved the highest AUC value of 0.90 in distinguishing PDAC from normal controls (Figure 4C).

### Characterization of EVs isolated from plasma

**Figure 5:**
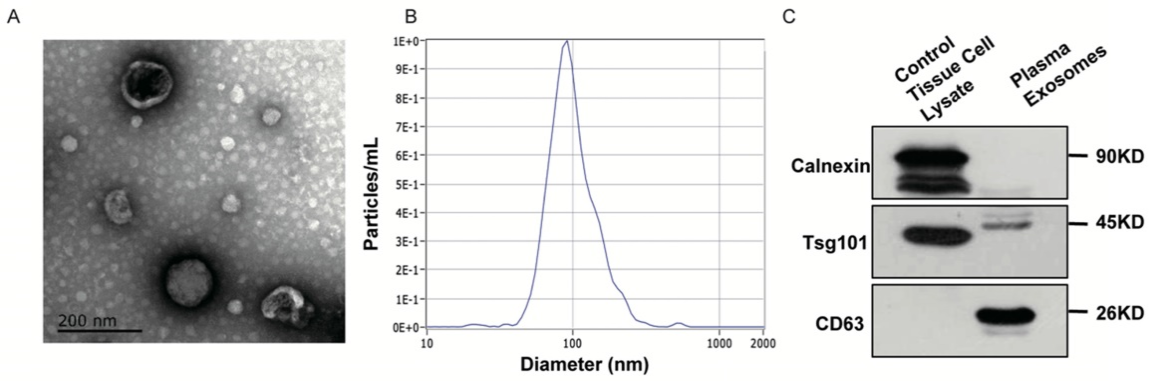
** Identification of EVs isolated in patients' plasma. A.** TEM revealed the external features of EVs isolated from plasma. The exosomes were oval or bowl-shaped capsules without the nucleus.** B.** NTA demonstrated EVs isolated from patients' plasma were 75-200nm in diameter.** C.** Verification of characteristic markers of EVs by Western Blot. Enrichment of two positive markers (TSG101 and CD63) were detected while the negative marker (calnexin) was absent in the isolated EVs samples.

In order to further evaluate the expression of EVs-derived RNAs in PDAC patients and non-cancerous individuals, we isolated EVs from plasma samples and characterized them according to the MISEV2018 guideline. We obtained images of oval or bowl-shaped microvesicles with diameters of 50-100 nm by TEM (Figure 5A). NTA was also performed with the peak value of diameters in 100nm (Figure 5B). We further verified distinctive markers for extracellular vesicles by Western Blot. Enrichment of two positive markers (TSG101 and CD63) were observed (Figure 5C). On the contrary, no protein band was detected around molecular weight of calnexin which was considered as a negative marker (Figure 5C).

### External validation by RT-qPCR data in our own cohort

**Figure 6:**
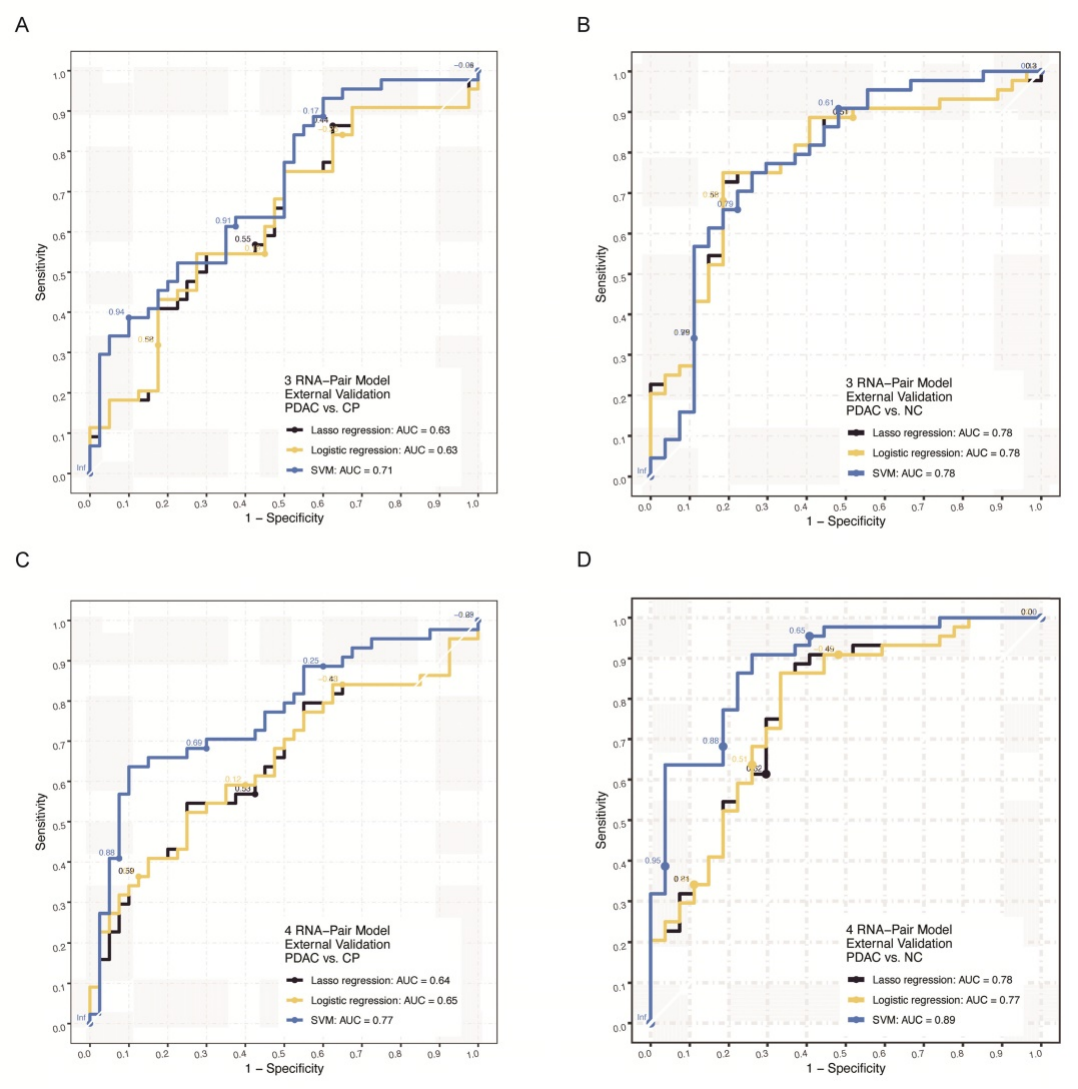
** External q-PCR verification of RNA-pair based models. A.** The ROC of models constructed by 3 RNA-pair with different algorithms in external RT-qPCR verification (PDAC vs. CP). **B.** The ROC of models constructed by 3 RNA-pair with different algorithms in external RT-qPCR verification (PDAC vs. NC). **C.** The ROC of models constructed by 4 RNA-pair with different algorithms in external RT-qPCR verification (PDAC vs. CP). **D.** The ROC of models constructed by 4 RNA-pair with different algorithms in external RT-qPCR verification (PDAC vs. NC). PADC: pancreatic ductal adenocarcinoma; CP: chronic pancreatitis; NC: normal control.

We further verified those signatures by RT-qPCR in an independent cohort of our own cohort including 111 samples (Clinical data was shown in Table 1). For 3 RNA pair model, SVM algorithm achieved the highest AUC of 0.71 in distinguishing PDAC from chronic pancreatitis patients and 0.78 in distinguishing PDAC from health individuals (Figure 6A-B, Figure S2B). For 4 RNA pair model, the SVM algorithm achieved the highest AUC of 0.77 in distinguishing PDAC from chronic pancreatitis patients and 0.89 in distinguishing PDAC from health individuals (Figure 6C-D, Figure S2C).

## Discussion

Detection of PDAC at a resectable stage is challenging to gastroenterologists^14^, ^19^. Many efforts have been made to evaluate EVs-derived RNAs as biomarkers in cancer diagnosis^7^, ^20^, especially in PDAC. Xian-yin Lai et al.^21^ developed an exosomal miRNA signature to predict PDAC, and found this signature has better diagnostic value comparing to CA19-9 and GPC1. Tetsuya Takikawa et al.^22^ focused on miRNAs in pancreatic stellate cell (PSC)-derived exosomes, and firstly clarified miRNA expression profile of PSC-related exosomes. They also confirmed the roles of PSC-exosome miRNAs in stimulating the proliferation or migration of PDAC cells. Shulin Yu et al.^23^ conducted the largest NGS study of EV-derived RNAs in PDAC with 501 participants, developed a diagnosis signature based on 8 EV-derived RNAs and exhibited high accuracy no matter in internal or external validation. They firstly characterized the plasma EV-derived RNA profile in PDAC. Their creative work had laid the groundwork of PDAC biomarker field and provided convincing evidence for EV-derived RNAs to be good PDAC diagnostic biomarkers.

However, there are still many problems preventing these signatures from clinical application. Testing cost is the major problem bothering physicians. Many researches were based on NGS or RNA chip data, however, these methods are not suitable for large-scale clinical application because of their high-cost. As a cost-effective tool, RT-qPCR could be widely used in the evaluation of EVs-derived RNAs, but the controversial normalization of RT-qPCR data generated from plasma EVs largely reduced the reproducibility across different studies as well as our enthusiasm in the development of liquid-biopsy based PDAC diagnostic tools.

Three regular ways were often used to normalize plasma EVs-derived RNAs. Traditional reference genes were often used to normalize RT-qPCR data, such as GAPDH and β-actin, which were considered stably expressed in somatic cells. However, these genes were proved to be differential expressed in plasma EVs. Moreover, during the isolation of EVs, different expression manners between target RNAs and internal reference RNAs result in variance in their relative expression levels and bring poor reproducibility. Consequently, they are not the ideal tool for data normalization in plasma EVs-derived RNAs quantification. Some researchers chose to screen more specific reference RNAs by many algorithms such as Normfinder, Genorm and DataAssist^24^-^29^. Dozens of reference RNAs were found in different diseases and various experiment situations; however due to insufficient sample volume of these studies and poor external validations, few of these reference RNAs were widely accepted by the scientific community. Spike-in exogenous RNAs to EVs such like cel-miR-39^30^-^32^ as external references to partially eliminate deviations from experimental processes was also a choice. However, sample specific deviations couldn't be corrected by those exogenous calibrators.

Here we proposed an RNA-pair ratio-based algorithm to quantitate plasma EVs-derived RNAs. The ratio of any two candidate RNAs were calculated in the same sample, then ratios between two RNAs were treated as new variables to construct a ratio-based prediction system by three machine learning algorithms. Subsequently, we validated these prediction models internally and externally, and found they were capable to predict PDAC occurrence with high AUC levels no matter in NGS data or RT-qPCR data.

The innovation points of this study lie on several aspects. Our major breakthrough is making the clinical application of diagnosis biomarker much easier. By using RT-qPCR method other than NGS or RNA chip, we reduced the cost of cancer screening. By using this normalizer-free algorithm, our model achieved high accuracy no matter in the NGS data or RT-qPCR data, which means we avoided the unstable factors induced by controversial reference genes and made RT-qPCR assays stable and reproducible. Cost-effective and high accuracy made our algorithm easier for clinical application. Moreover, three machine learning methods including lasso regression, SVM and logistic regression were simultaneously applied to assess the robustness of prediction model, making the result more convincing. Cross-platform property also enhanced the reproducibility of this research. Last but not least, our innovation is localized in methodology level and could be helpful to researchers intending to discover reliable biomarkers for other cancer types.

Mattia Boeri etc.^11^ firstly used RNA ratio signatures to predict patients' prognosis with minimum AUC level of 0.85. However, due to its sufficient sample volume and no mathematically proofs were given, it was still hard for researchers to understand this method. Deng etc.^33^ firstly mathematically verified RNA ratio methods. Their findings showed that RNA ratio could reflect true fold change between 2 RNAs by eliminating disturbance of various reference RNAs because expression levels of two RNAs were simultaneously measured under same conditions. Moreover, they also found that RNA ratios method was independent of spiked-in and internal controls, and was more robust than internal or external reference RNAs by comparing their diagnostic capacity, which gave this article a robust theoretical basis.

We identified 8 biomarkers to predict PDAC, i.e. FBXO7, MORF4L1, DDX17, TALDO1, AHNAK, TUBA1B, CD44, and SETD3. Among them, CD44 was proved to be involved in biological process as cell-cell interactions, cell adhesion and migration, which was also enriched in GO analysis of this study^34^, ^35^. In recent years, researchers found CD44 was a biomarker of PDAC cancer stem cells 36, and could reflect initiation and metastasis of PDAC, which made CD44 a potential biomarker to predict PDAC. AHNAK is a large scaffold protein and participates in focal adhesion and cell-substrate junction^37^, ^38^, which is also enriched in GO analysis. AHNAK is proved to be associated with poor prognosis of PDAC patients through epithelial-mesenchymal transition, and could be a biomarker to predict PDAC patients' outcome^39^. FBXO7 is a component of ubiquitin-protein ligase complex and plays vital roles in mediating ubiquitination and proteasomal degradation of proteins^40^-^42^. However, its roles in predicting PDAC remains unknown. Most of these biomarkers were related to tumorigenesis or metastasis. Biological or pathological functions of them were still unclear, further experiments would be performed to explore their roles in EVs and their relationship with PDAC.

## Conclusion

In conclusion, we firstly developed a qPCR-based, normalizer-free circulating EVs RNA classifier, which could well distinguish PDAC patients from noncancerous controls. After validation of both NGS data and RT-qPCR data of our independent cohort, we rigorously confirmed the robustness and cross-platform stability of this approach. The gene-pair focused methodology we established here would also be helpful to find more qPCR-based, normalizer-free models from the public available EVs RNA databases in the studies of other cancer types.

## Supplementary Material

Supplementary figures and tables.

## Figures and Tables

**Table 1:** Clinical characteristics of enrolled patients

	GSE133684			External validation samples		
	**NC**	**CP**	**PDAC**	**NC**	**CP**	**PDAC**
Number	117	100	284	27	40	44
Age Range (y)	41-91	8-78	31-84	50-73	30-89	20-89
**Gender**						
Male	71	73	167	12	26	22
Female	46	27	117	15	14	22
Serum CA19-9(U/ml)	NA	2-1200	0.6-1200	NA	0.8-673.9	0.8-1972
**Clinical Stage**						
I			59			10
II			77			13
III			42			7
IV			106			11

**Table 2:** RNA models establishing

Gene-pair models	RNA-pairs	coefficient	Lambda	AUC
2-RNA-pair model	FBXO7/MORF4L1	-0.005	0.26	0.901
	DDX17/TALDO1	-0.095		
3-RNA-pair model	FBXO7/MORF4L1	-0.020	0.25	0.919
	DDX17/TALDO1	-0.193		
	AHNAK/TUBA1B	-0.002		
4-RNA-pair model	FBXO7/MORF4L1	-0.037	0.24	0.935
	DDX17/TALDO1	-0.277		
	AHNAK/TUBA1B	-0.017		
	CD44/SETD3	-0.003		

## Abbreviations

PDAC: Pancreatic ductal adenocarcinoma
PC: Pancreatic cancer
EVs: extracellular vesicles
NGS: next generation sequencing
RT-qPCR: Real-time quantitative PCR
CP: chronic pancreatitis patients
NC: normal controls
DE: Differentially expressed
BP: biological processes
CC: cellular components
MF: molecular features
GO: Gene Ontology
SVM: support vector machine
ROC: Receiver operator characteristic
AUC: area under the curve
UC: ultracentrifugation
NTA: Nanoparticle tracking analysis
TEM: Transmission electron microscopy

## References

[1] Rahib L, Smith BD, Aizenberg R (2014). Projecting cancer incidence and deaths to 2030: the unexpected burden of thyroid, liver, and pancreas cancers in the United States. *Cancer Res*.

[2] Sikdar N, Saha G, Dutta A (2018). Genetic Alterations of Periampullary and Pancreatic Ductal Adenocarcinoma: An Overview. *Curr Genomics*.

[3] Li J-T, Wang Y-P, Yin M (2019). Metabolism remodeling in pancreatic ductal adenocarcinoma. *Cell Stress*.

[4] Lennon KM, Wakefield DL, Maddox AL (2019). Single molecule characterization of individual extracellular vesicles from pancreatic cancer. *J Extracell Vesicles*.

[5] Giulietti M, Righetti A, Principato G (2018). LncRNA co-expression network analysis reveals novel biomarkers for pancreatic cancer. *Carcinogenesis*.

[6] Crossland RE, Norden J, Bibby LA (2016). Evaluation of optimal extracellular vesicle small RNA isolation and qRT-PCR normalisation for serum and urine. *J Immunol Methods*.

[7] Min L, Zhu S, Chen L (2019). Evaluation of circulating small extracellular vesicles derived miRNAs as biomarkers of early colon cancer: a comparison with plasma total miRNAs. *J Extracell Vesicles*.

[8] Liu X, Zhang L, Cheng K (2014). Identification of suitable plasma-based reference genes for miRNAome analysis of major depressive disorder. *J Affect Disord*.

[9] Xiang M, Zeng Y, Yang R (2014). U6 is not a suitable endogenous control for the quantification of circulating microRNAs. *Biochem Biophys Res Commun*.

[10] Donati S, Ciuffi S, Brandi ML (2019). Human Circulating miRNAs Real-time qRT-PCR-based Analysis: An Overview of Endogenous Reference Genes Used for Data Normalization. *Int J Mol Sci*; 20. Epub ahead of print 5 September.

[11] Madadi S, Schwarzenbach H, Lorenzen J (2019). MicroRNA expression studies: challenge of selecting reliable reference controls for data normalization. *Cell Mol Life Sci CMLS*.

[12] Occhipinti G, Giulietti M, Principato G (2016). The choice of endogenous controls in exosomal microRNA assessments from biofluids. *Tumour Biol J Int Soc Oncodevelopmental Biol Med*.

[13] Schaefer A, Jung M, Miller K (2010). Suitable reference genes for relative quantification of miRNA expression in prostate cancer. *Exp Mol Med*.

[14] Yu S, Li Y, Liao Z (2019). Plasma extracellular vesicle long RNA profiling identifies a diagnostic signature for the detection of pancreatic ductal adenocarcinoma. *Gut*.

[15] Ritchie ME, Phipson B, Wu D (2015). limma powers differential expression analyses for RNA-sequencing and microarray studies. *Nucleic Acids Res*.

[16] Yu G, Wang L-G, Han Y (2012). clusterProfiler: an R package for comparing biological themes among gene clusters. *Omics J Integr Biol*.

[17] Friedman J, Hastie T, Tibshirani R (2010). Regularization Paths for Generalized Linear Models via Coordinate Descent. *J Stat Softw*.

[18] Sachs MC (2017). plotROC: A Tool for Plotting ROC Curves. *J Stat Softw*; 79. Epub ahead of print August.

[19] Bailey P, Chang DK, Nones K (2016). Genomic analyses identify molecular subtypes of pancreatic cancer. *Nature*.

[20] Shah R, Patel T, Freedman JE (2018). Circulating Extracellular Vesicles in Human Disease. *N Engl J Med*.

[21] Lai X, Wang M, McElyea SD (2017). A microRNA signature in circulating exosomes is superior to exosomal glypican-1 levels for diagnosing pancreatic cancer. *Cancer Lett*.

[22] Takikawa T, Masamune A, Yoshida N (2017). Exosomes Derived From Pancreatic Stellate Cells: MicroRNA Signature and Effects on Pancreatic Cancer Cells. *Pancreas*.

[23] Yu S, Li Y, Liao Z (2020). Plasma extracellular vesicle long RNA profiling identifies a diagnostic signature for the detection of pancreatic ductal adenocarcinoma. *Gut*.

[24] Ragni E, Perucca Orfei C, De Luca P (2019). Identification of miRNA Reference Genes in Extracellular Vesicles from Adipose Derived Mesenchymal Stem Cells for Studying Osteoarthritis. *Int J Mol Sci*; 20. Epub ahead of print 5 March.

[25] Guo R, Guo H, Zhang Q (2018). Evaluation of reference genes for RT-qPCR analysis in wild and cultivated Cannabis. *Biosci Biotechnol Biochem*.

[26] Brown AJ, Gibson S, Hatton D (2018). Transcriptome-Based Identification of the Optimal Reference CHO Genes for Normalisation of qPCR Data. *Biotechnol J*; 13. Epub ahead of print January.

[27] Augustyniak J, Lenart J, Lipka G (2019). Reference Gene Validation via RT-qPCR for Human iPSC-Derived Neural Stem Cells and Neural Progenitors. *Mol Neurobiol*.

[28] Gao Z, Deng W, Zhu F (2019). Reference gene selection for quantitative gene expression analysis in black soldier fly (Hermetia illucens). *PloS One*.

[29] Wang Q, Ishikawa T, Michiue T (2012). Stability of endogenous reference genes in postmortem human brains for normalization of quantitative real-time PCR data: comprehensive evaluation using geNorm, NormFinder, and BestKeeper. *Int J Legal Med*.

[30] Perge P, Decmann Á, Pezzani R (2018). Analysis of circulating extracellular vesicle-associated microRNAs in cortisol-producing adrenocortical tumors. *Endocrine*.

[31] Sohn W, Kim J, Kang SH (2015). Serum exosomal microRNAs as novel biomarkers for hepatocellular carcinoma. *Exp Mol Med*.

[32] Madadi S, Soleimani M (2019). Comparison of miR-16 and cel-miR-39 as reference controls for serum miRNA normalization in colorectal cancer. *J Cell Biochem*.

[33] Deng Y, Zhu Y, Wang H (2019). Ratio-Based Method To Identify True Biomarkers by Normalizing Circulating ncRNA Sequencing and Quantitative PCR Data. *Anal Chem*.

[34] Wu C, Thalhamer T, Franca RF (2014). Galectin-9-CD44 interaction enhances stability and function of adaptive regulatory T cells. *Immunity*.

[35] Naujokas MF, Morin M, Anderson MS (1993). The chondroitin sulfate form of invariant chain can enhance stimulation of T cell responses through interaction with CD44. *Cell*.

[36] Ishiwata T, Matsuda Y, Yoshimura H (2018). Pancreatic cancer stem cells: features and detection methods. *Pathol Oncol Res POR*.

[37] Chen B, Wang J, Dai D (2017). AHNAK suppresses tumour proliferation and invasion by targeting multiple pathways in triple-negative breast cancer. *J Exp Clin Cancer Res CR*.

[38] Zhang Z, Liu X, Huang R (2019). Upregulation of nucleoprotein AHNAK is associated with poor outcome of pancreatic ductal adenocarcinoma prognosis via mediating epithelial-mesenchymal transition. *J Cancer*.

[39] Zhang Z, Liu X, Huang R (2019). Upregulation of nucleoprotein AHNAK is associated with poor outcome of pancreatic ductal adenocarcinoma prognosis via mediating epithelial-mesenchymal transition. *J Cancer*.

[40] Burchell VS, Nelson DE, Sanchez-Martinez A (2013). The Parkinson's disease-linked proteins Fbxo7 and Parkin interact to mediate mitophagy. *Nat Neurosci*.

[41] Chang Y-F, Cheng C-M, Chang L-K (2006). The F-box protein Fbxo7 interacts with human inhibitor of apoptosis protein cIAP1 and promotes cIAP1 ubiquitination. *Biochem Biophys Res Commun*.

[42] Hsu J-M, Lee Y-CG, Yu C-TR (2004). Fbx7 functions in the SCF complex regulating Cdk1-cyclin B-phosphorylated hepatoma up-regulated protein (HURP) proteolysis by a proline-rich region. *J Biol Chem*.

