# Supplementary material for: Detection of Pancreatic Ductal Adenocarcinoma by A qPCR-based Normalizer-free Circulating Extracellular Vesicles RNA Signature
Source: J Cancer. 2021 Jan 1;12(5):1445–54. doi: 10.7150/jca.50716 (PMC7847660; doi:10.7150/jca.50716)
Supplement: Supplementary file 1 — Supplementary figures and tables. [file jcav12p1445s1.pdf]

Figure S1. GO analysis of DE genes.

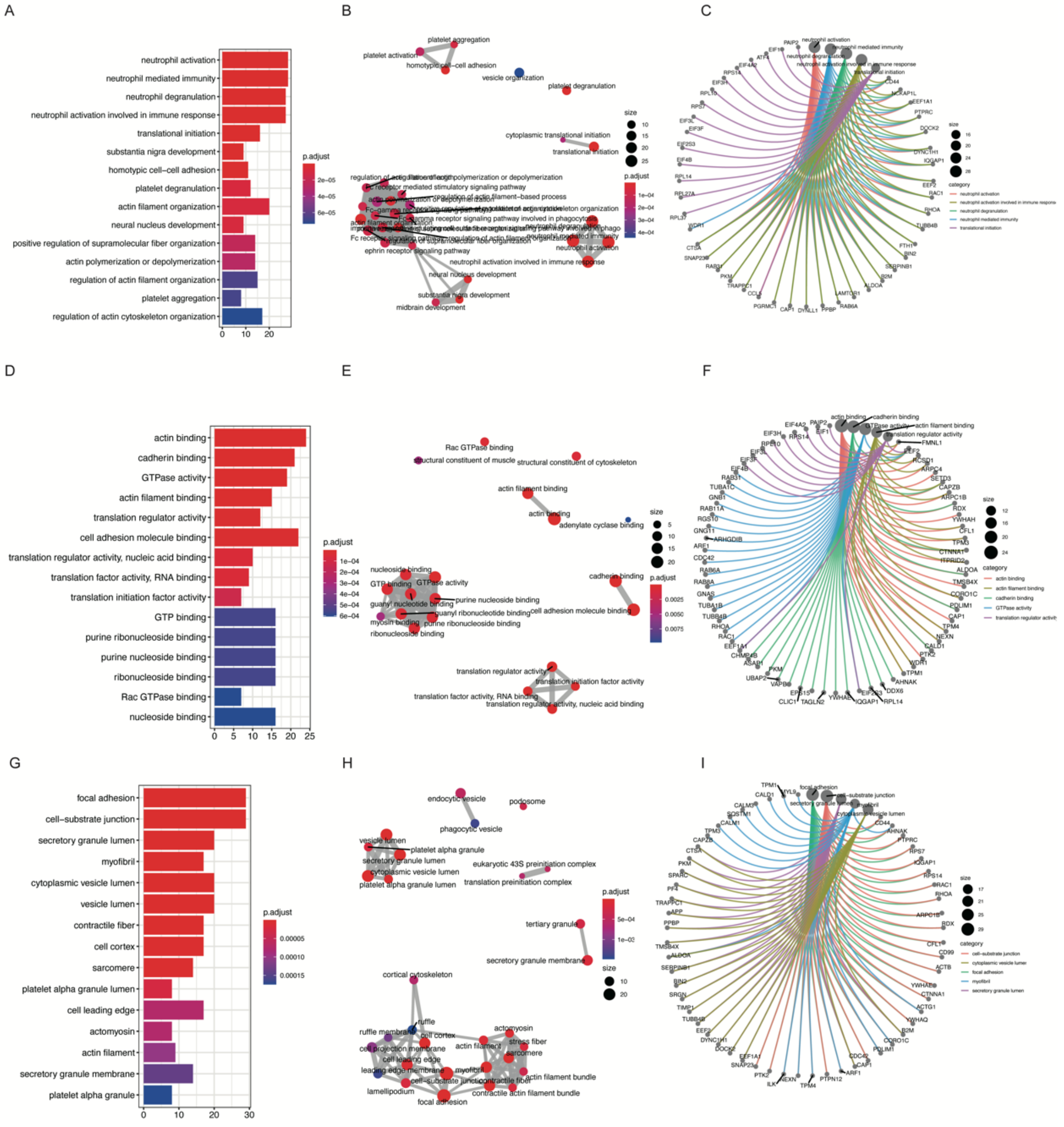

A. Bar plot to show DE RNAs enriched biological processes. Neutrophil activation and neutrophil mediated immunity were significantly enriched. B. Relationship of enriched-biological processes. C. Genes involved in enriched biological processes. D. Bar plot showing DE RNAs enriched molecular function. Actin binding and cell adhesion molecule binding were significantly enriched. E. Relationship of enriched-molecular functions. F. Genes involved in enriched molecular functions. G. Bar plot to show DE RNAs enriched cellular components. H. Relationship of enriched-cellular components. I. Genes involved in enriched cellular components.

Figure S2. Model establishment and verification.

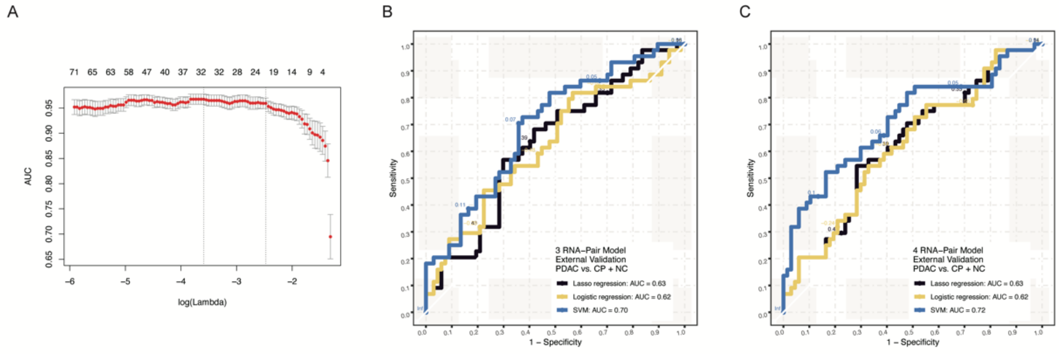

A. Relationship between lambda and AUC in Lasso regression. B. The ROC of models constructed by 3 RNA-pair with different algorithms in external RT-qPCR verification (PDAC vs. NC + CP). C. The ROC of models constructed by 4 RNA-pair with different algorithms in external RT-qPCR verification (PDAC vs. NC + CP).

Table S1. Primer sequence used in RT-qPCR assay

Primer name	Primer sequence	Base number
CD44-F	CATGGACAAGTTTTGGTGGCA	21
CD44-R	CGTGGAATACACCTGCAAAGC	21
CD44-P	ACTCTGCCTCGTGCCGCTGA	20
FBXO7-F	ATCTGCGTGATTTTCGAGACA	21
FBXO7-R	CATCACAAACCGCCCTTTC	19
FBXO7-P	ACTGTCAGAGTTCAAGACACAGATTGGA	28
MORF4L1-F	AGTATGCAGAGGGGAAGATGAG	22
MORF4L1-R	TGGTACTGCCACCATCTCC	19
MORF4L1-P	TGCCCCAGGAAAGAAGACATCTGGTCT	27
SETD3-F	AACACAGCTCGACAGTACGC	20
SETD3-R	GCCCACCTGTAGTCCTCGTA	20
SETD3-P	TCATCCAGACCCATCCTCATGCCA	24
TALDO1-F	CGTCAGAGGATGGAGTCCG	19
TALDO1-R	TCCTGGGGCTTGTACTCGT	19
TALDO1-P	AGCTCAAGCAGTTCACCACCGT	22
TUBA1B-F	CTCCTAATCCCTAGCCACTATGC	23
TUBA1B-R	TGCCAATCTGGACACCAGC	19
TUBA1B-P	TGAGTGCATCTCCATCCACGTTGGC	25
DDX17-F	ACCACCAGCCATACTTGGA	19
DDX17-R	GGGCAAGCTCTCTGGTAGG	19
DDX17-P	ACTAGACAGATTGGGCCATCTCCCCT	26
AHNAK-F	CAGGAGGTGACGCAGAACTC	20
AHNAK-R	ATGGTGGCACCCACAATCTG	20
AHNAK-P	TGGGGTGGTCAAGGAGGGGGA	21

* F means forward primer. R means reverse primer. P means probe.

Table S2. DE genes coefficient

	logFC	AveExpr	t	P.Value	adj.P.Val	B
TCF7	48.757507	70.8957057	10.7989053	5.38E-23	1.39E-18	41.0863351
BTG1	65.3076316	163.388013	10.1863977	5.73E-21	7.38E-17	36.606254
CD44	93.3218941	225.859549	10.0445943	1.66E-20	1.43E-16	35.585328
AHNAK	546.695986	1067.56053	9.7562181	1.41E-19	9.11E-16	33.5296092
STK17B	87.7919868	267.076635	9.68793846	2.34E-19	1.21E-15	33.0470435
FBXO7	184.894928	332.219956	9.07498102	1.97E-17	8.48E-14	28.7917677
TNRC6B	35.64705	82.7047041	8.98173829	3.82E-17	1.41E-13	28.1572414
BACH2	18.0854592	27.2725701	8.69303329	2.90E-16	9.33E-13	26.2154578
PAX5	36.8914844	56.747935	8.66117508	3.61E-16	1.03E-12	26.0033616

DYRK2	19.4772838	41.2781726	8.63720371	4.27E-16	1.10E-12	25.8440637
MARF1	54.5638569	178.685291	8.41998974	1.90E-15	4.45E-12	24.4122172
IKZF3	56.9888652	138.462337	8.40421081	2.12E-15	4.55E-12	24.3090327
CAMK1D	20.7186221	50.3834737	8.33397868	3.41E-15	6.77E-12	23.8511439
ITGA4	40.5331851	83.9750389	8.31316427	3.93E-15	7.24E-12	23.7158789
FAM102A	22.5461711	44.0140801	8.29785347	4.36E-15	7.49E-12	23.6165085
DDX6	68.2159127	285.936245	8.2705865	5.24E-15	8.45E-12	23.4398108
SRCAP	22.061791	58.3771431	8.11573409	1.48E-14	2.25E-11	22.4429828
TXNIP	247.559862	942.429715	8.07981509	1.89E-14	2.70E-11	22.2134014
ARID1A	48.5250982	202.269559	7.98365402	3.57E-14	4.84E-11	21.6018641
RALGPS2	37.3763865	71.3988092	7.89347964	6.47E-14	8.17E-11	21.0325401

SESN3	71.461084	156.743403	7.88917945	6.66E-14	8.17E-11	21.0054916
KLF13	43.1320393	142.207362	7.80237118	1.18E-13	1.32E-10	20.4614539
LOC105370939	43.1320393	142.207362	7.80237118	1.18E-13	1.32E-10	20.4614539
NCKAP1L	44.0768584	139.546339	7.74402897	1.72E-13	1.78E-10	20.0979662
BCL11B	21.1700512	45.526727	7.74348582	1.72E-13	1.78E-10	20.0945905
IL7R	46.4689085	85.7917163	7.68866513	2.46E-13	2.40E-10	19.7546507
LBH	49.8746529	144.48379	7.68541091	2.51E-13	2.40E-10	19.7345204
IL16	34.4542683	123.388056	7.60166002	4.31E-13	3.97E-10	19.2183409
SLC38A1	31.5883517	78.5748589	7.51358002	7.57E-13	6.73E-10	18.6794597
BCL9L	29.8906252	55.5635768	7.40581482	1.50E-12	1.26E-09	18.0257775
ELMSAN1	22.1780673	59.8994642	7.40396799	1.52E-12	1.26E-09	18.0146296

CHMP7	17.618379	45.7780054	7.37821827	1.78E-12	1.44E-09	17.8593916
SRRM2	119.231978	281.484035	7.3671275	1.91E-12	1.49E-09	17.7926397
RPL37	143.784561	422.725119	7.32223212	2.54E-12	1.88E-09	17.5231157
PRPF8	47.49208	178.761878	7.32124582	2.55E-12	1.88E-09	17.5172069
EEF1A1	1855.65122	7047.03867	7.30178316	2.88E-12	2.01E-09	17.4007198
CAMK4	11.8723779	17.1960648	7.30146894	2.89E-12	2.01E-09	17.3988408
CD37	65.0175641	265.436521	7.29647978	2.98E-12	2.02E-09	17.3690144
AAK1	25.2752919	68.2240326	7.27759301	3.35E-12	2.18E-09	17.2562291
ELK4	49.7799387	199.108628	7.27586723	3.39E-12	2.18E-09	17.2459332
FAM117B	16.2796804	42.4666486	7.24752119	4.04E-12	2.54E-09	17.0770585
KMT2D	43.0650382	89.0015522	7.22425224	4.67E-12	2.87E-09	16.9387652

FOXP1	33.5608974	130.362555	7.21717292	4.88E-12	2.93E-09	16.8967509
WDFY4	31.5281436	64.0517348	7.17674242	6.27E-12	3.55E-09	16.6573423
BMF	8.8452367	16.4117597	7.1759242	6.31E-12	3.55E-09	16.6525066
BNIP3L	695.162157	1351.69348	7.17502069	6.34E-12	3.55E-09	16.6471674
RPL27A	177.783028	682.436035	7.15305369	7.26E-12	3.98E-09	16.5174966
BCL11A	15.0047616	34.4557716	7.1475768	7.51E-12	4.03E-09	16.485209
FOXO1	17.4759571	48.3709853	7.14137143	7.81E-12	4.11E-09	16.4486473
BLK	11.7645252	20.4227396	7.13109038	8.32E-12	4.29E-09	16.3881199
SEC16A	23.3136941	64.6618934	7.12554864	8.60E-12	4.35E-09	16.355519
LANCL1	10.9264771	23.0874854	7.09501511	1.04E-11	5.15E-09	16.1762091
SETD1B	17.927158	37.9932412	7.08686361	1.09E-11	5.31E-09	16.1284286

ARPC4-TTLL3	-36.547618	90.6603451	-7.0827228	1.12E-11	5.34E-09	16.1041715
CYLD	32.2422314	117.063454	7.03510781	1.50E-11	7.02E-09	15.8259455
MS4A1	20.459955	41.6253784	7.01351397	1.71E-11	7.87E-09	15.7001963
SPIB	9.06781276	17.4771871	7.00680007	1.78E-11	8.05E-09	15.6611535
HLA-DPA1	42.4667623	116.578612	6.97535807	2.16E-11	9.58E-09	15.4786579
LEF1	24.2121934	51.951519	6.95629108	2.42E-11	1.06E-08	15.3682684
GAPDH	-344.81775	1059.79739	-6.9460393	2.57E-11	1.11E-08	15.3090025
ZFP36L2	41.6588209	119.601105	6.9178222	3.05E-11	1.29E-08	15.1461949
CBLB	15.5028307	39.4134193	6.91106166	3.18E-11	1.32E-08	15.1072569
KIF21B	24.9047709	59.848994	6.8937261	3.53E-11	1.44E-08	15.0075333

TNFRSF13C	11.063328	17.4315163	6.88835399	3.65E-11	1.46E-08	14.9766658
BAZ2A	35.2608574	138.10719	6.88707686	3.67E-11	1.46E-08	14.9693301
KLHL36	11.2610636	27.4030021	6.87532267	3.94E-11	1.54E-08	14.9018599
SON	65.8808537	266.360449	6.862654	4.26E-11	1.64E-08	14.8292316
MLLT6	44.9687015	123.2468	6.82253288	5.41E-11	2.03E-08	14.5998463
IQSEC1	41.0125343	132.470439	6.82147244	5.45E-11	2.03E-08	14.5937964
SSR2	16.966792	49.6276393	6.80504954	6.01E-11	2.21E-08	14.5001869
ZBTB20	20.9982447	61.0070212	6.78846291	6.63E-11	2.41E-08	14.405807
ZNF91	40.5774495	118.721291	6.78615883	6.73E-11	2.41E-08	14.3927095
TMC8	16.0686456	21.7376301	6.72288308	9.79E-11	3.46E-08	14.03426
RPL14	165.808062	552.692113	6.69402358	1.16E-10	4.05E-08	13.8715734

WDR82	29.4016115	122.633708	6.66715394	1.36E-10	4.68E-08	13.7205565
RASSF2	46.3991664	181.379187	6.61347047	1.86E-10	6.33E-08	13.4201499
AKNA	82.1424878	277.345399	6.55983928	2.55E-10	8.54E-08	13.1217951
GNG7	5.9461979	9.82309419	6.55370254	2.64E-10	8.74E-08	13.0877687
CTC1	10.8464989	20.2818767	6.50475412	3.51E-10	1.15E-07	12.8171966
ARPC4	-46.08541	143.73327	-6.4896583	3.83E-10	1.23E-07	12.734051
ETS1	103.908628	433.227473	6.48586946	3.92E-10	1.25E-07	12.7132049
MAP3K1	39.6571537	153.654132	6.47698128	4.12E-10	1.30E-07	12.6643374
NSUN3	14.7914537	22.7214677	6.46089078	4.52E-10	1.40E-07	12.5759966
EIF4B	164.342297	800.55662	6.44556538	4.94E-10	1.52E-07	12.4920067

DENND4B	13.7161717	30.674591	6.43938087	5.12E-10	1.55E-07	12.4581545
GGA2	20.7132603	64.6990133	6.43666283	5.20E-10	1.56E-07	12.4432843
CRTC3	12.4049546	32.0963603	6.43129862	5.36E-10	1.57E-07	12.4139507
MGAT4A	11.8340118	27.2937577	6.43113491	5.37E-10	1.57E-07	12.4130558
FMNL3	17.9969756	50.3012361	6.42164199	5.67E-10	1.64E-07	12.36119
PRDM2	21.0991506	94.4916126	6.41782821	5.79E-10	1.66E-07	12.3403689
SFT2D2	14.2830677	34.2811394	6.40259956	6.32E-10	1.79E-07	12.2573199
REL	16.3637967	49.4509804	6.4008075	6.39E-10	1.79E-07	12.2475566
HDGF	-90.455699	237.091252	-6.3980974	6.49E-10	1.80E-07	12.2327957
AFF3	16.021434	32.5660033	6.38484042	7.00E-10	1.92E-07	12.1606556

RAC1	-80.196748	244.296587	-6.3798864	7.20E-10	1.94E-07	12.1337257
MYL6	-646.80202	1120.25674	-6.3790675	7.23E-10	1.94E-07	12.1292759
INPP5D	45.8126563	175.535178	6.36463099	7.85E-10	2.09E-07	12.0508962
MORF4L1	-55.726246	196.507188	-6.361847	7.98E-10	2.10E-07	12.0357965
BTBD10	-16.483462	46.6936169	-6.341912	8.94E-10	2.32E-07	11.9278159
SIK3	16.0630968	60.2497933	6.34029053	9.02E-10	2.32E-07	11.9190441
PRRC2B	32.1266346	110.158272	6.33814045	9.13E-10	2.33E-07	11.9074152
SETD3	-42.092363	163.71782	-6.3283294	9.65E-10	2.44E-07	11.8543885

UBE2Q1	-29.603483	90.3014352	-6.3205856	1.01E-09	2.52E-07	11.8125782
NUCB2	-26.264626	63.5335414	-6.3088341	1.08E-09	2.67E-07	11.749202
PTMA	-1091.161	2331.5514	-6.2868784	1.22E-09	3.00E-07	11.631031
ATM	45.3640335	167.032344	6.27727625	1.29E-09	3.13E-07	11.5794467
MSL2	14.9106503	48.0196111	6.27208543	1.33E-09	3.19E-07	11.5515854
EIF2S3	59.8505422	238.746091	6.27072613	1.34E-09	3.19E-07	11.5442923
PHF14	-30.76049	90.0435524	-6.2679006	1.36E-09	3.21E-07	11.5291364
EML3	6.31431813	12.8426569	6.26286223	1.40E-09	3.26E-07	11.5021231

ARNTL2	-10.392603	15.3846725	-6.2622026	1.40E-09	3.26E-07	11.4985878
DERA	-27.942988	42.5189334	-6.2430619	1.56E-09	3.60E-07	11.3961224
PPP1R16B	22.3223182	70.3775412	6.23992144	1.59E-09	3.63E-07	11.3793328
OGT	31.8111235	53.148524	6.22346618	1.74E-09	3.93E-07	11.2914648
TBCEL	26.2855728	76.0792098	6.22254508	1.75E-09	3.93E-07	11.2865515
DNAJC2	-33.392505	77.9173963	-6.2185226	1.79E-09	3.96E-07	11.2651012
COP1	-20.405355	60.0921597	-6.2182116	1.80E-09	3.96E-07	11.2634431
TALDO1	-137.69509	326.40663	-6.2125395	1.85E-09	4.05E-07	11.233215

CAPZB	-80.273804	277.45381	-6.2010539	1.98E-09	4.26E-07	11.1720686
EHBP1	-36.441574	91.0761977	-6.2004675	1.98E-09	4.26E-07	11.168949
POLR2A	36.5296409	146.298912	6.19657655	2.03E-09	4.32E-07	11.1482554
RBL2	32.1621482	158.029772	6.19461544	2.05E-09	4.33E-07	11.1378291
RHOA	-218.4671	715.333343	-6.1911302	2.09E-09	4.38E-07	11.1193059
ATF4	-149.53465	292.089787	-6.1813437	2.21E-09	4.55E-07	11.0673353
SMS	-36.593717	95.6491896	-6.1812849	2.21E-09	4.55E-07	11.0670232
ANP32B	-295.62157	787.666154	-6.1801549	2.22E-09	4.55E-07	11.0610269

SMYD3	-18.741213	27.0239599	-6.1782617	2.25E-09	4.56E-07	11.0509813
SMCR8	15.9893466	51.6076941	6.17478278	2.29E-09	4.61E-07	11.0325289
COBLL1	16.4305343	39.2777097	6.16773251	2.38E-09	4.76E-07	10.9951574
DAP	-168.67844	257.325526	-6.1647634	2.42E-09	4.80E-07	10.9794288
CYFIP2	62.0868215	265.386288	6.16335447	2.44E-09	4.80E-07	10.9719669
FNBP1	42.5646077	197.368858	6.15915063	2.50E-09	4.88E-07	10.9497109
RCAN3	24.9345853	74.0090553	6.15355183	2.58E-09	4.99E-07	10.9200875
KIAA1147	15.4283369	43.415886	6.15175389	2.60E-09	4.99E-07	10.9105789
TUBB4B	-73.805082	147.4053	-6.1498369	2.63E-09	4.99E-07	10.900443

TIMP1	-239.68043	273.548489	-6.1497733	2.63E-09	4.99E-07	10.9001069
MDM4	16.9734002	41.7768832	6.14750079	2.67E-09	5.02E-07	10.8880942
TMEM56-RWDD3	10.3393465	14.5541071	6.14335778	2.73E-09	5.10E-07	10.8662029
FTH1	-7745.9138	11540.5452	-6.1398931	2.78E-09	5.16E-07	10.8479047
ICA1	-25.028571	40.75871	-6.1359539	2.84E-09	5.23E-07	10.8271094
NUFIP2	23.6925029	88.6459311	6.13194638	2.91E-09	5.31E-07	10.805964
ZCCHC17	-121.0764	168.525873	-6.1094433	3.29E-09	5.98E-07	10.6874231
OSBPL10	9.45881452	18.8242493	6.10622842	3.35E-09	6.04E-07	10.6705149
EIF3F	37.787388	148.085395	6.1042621	3.39E-09	6.07E-07	10.6601767

SZT2	9.6533154	16.299595	6.1029956	3.41E-09	6.07E-07	10.6535192
FOXK1	11.5810072	29.5676821	6.09592368	3.55E-09	6.26E-07	10.6163643
HSPB1	-43.335893	101.807189	-6.0946944	3.57E-09	6.26E-07	10.6099092
JADE2	21.2027801	69.4104562	6.08993072	3.67E-09	6.39E-07	10.5849038
EIF3L	60.3853886	263.225357	6.08734432	3.72E-09	6.39E-07	10.5713336
WIPI1	-23.68295	45.2784381	-6.087337	3.72E-09	6.39E-07	10.5712951
CCNB1	-13.440335	24.6623371	-6.0858294	3.75E-09	6.41E-07	10.5633871
ARPC1B	-261.18776	520.961574	-6.0843193	3.78E-09	6.42E-07	10.5554674
BCAP31	-78.357019	100.576669	-6.0807998	3.86E-09	6.50E-07	10.5370156

RAB32	-49.987462	72.9931696	-6.077242	3.93E-09	6.58E-07	10.5183709
LUC7L2	-75.714993	132.978363	-6.0704879	4.08E-09	6.79E-07	10.4829997
SRGN	-230.63658	303.029175	-6.0632516	4.25E-09	7.02E-07	10.4451359
RHOC	-24.558034	87.148694	-6.049834	4.57E-09	7.51E-07	10.3750204
TUBA1B	-183.00828	592.11784	-6.0485991	4.61E-09	7.51E-07	10.3685733
RDX	-42.628636	132.742822	-6.0312093	5.07E-09	8.22E-07	10.2778913
CETN2	-33.995221	45.8685883	-6.0274118	5.17E-09	8.34E-07	10.2581152

CIITA	19.0907657	39.1489507	6.01801652	5.45E-09	8.72E-07	10.2092288
RNF44	18.8945664	51.6064464	6.01419814	5.56E-09	8.82E-07	10.1893774
OPA1	40.2889368	111.873195	6.01376845	5.58E-09	8.82E-07	10.187144
LCN2	-66.651761	71.5333866	-6.0122774	5.62E-09	8.83E-07	10.179395
UBC	-150.87499	772.276951	-6.0111659	5.66E-09	8.83E-07	10.1736199
INPP4A	20.1415475	62.3513229	6.00386388	5.89E-09	9.14E-07	10.1356989
PTBP3	43.0741405	177.683568	5.99168736	6.29E-09	9.71E-07	10.0725423
SMIM14	19.5174948	57.0349749	5.98688876	6.46E-09	9.89E-07	10.0476802
CNOT1	46.5798311	214.241103	5.98606463	6.49E-09	9.89E-07	10.0434118
SIPA1L3	16.8609544	48.5005063	5.98439208	6.55E-09	9.91E-07	10.0347507

PLEKHA2	29.1989079	119.703173	5.98366763	6.57E-09	9.91E-07	10.0309998
HIVEP2	22.5079491	84.4758318	5.97635155	6.84E-09	1.02E-06	9.99313954
MITF	-19.654953	25.3152434	-5.9696787	7.09E-09	1.06E-06	9.95863907
CXCR5	7.60798447	11.8292135	5.96652816	7.21E-09	1.07E-06	9.94236023
YWHAH	-385.36804	541.132018	-5.9596098	7.49E-09	1.10E-06	9.90663613
UBE2N	11.9410075	37.9963089	5.94778026	7.99E-09	1.17E-06	9.8456268
EP400	18.6553171	53.5586809	5.94569873	8.08E-09	1.18E-06	9.8349012
TMEM131L	11.7548638	30.0358587	5.94041703	8.31E-09	1.20E-06	9.80769898
CFL1	-203.44827	696.977584	-5.9341491	8.60E-09	1.24E-06	9.77544181

HIST1H2BD	-71.440515	115.760409	-5.9324838	8.68E-09	1.24E-06	9.76687576
CD99	-273.41534	357.632506	-5.9321219	8.70E-09	1.24E-06	9.76501455
C9orf3	-12.895527	24.0176117	-5.9276719	8.91E-09	1.26E-06	9.7421351
MYL12B	-159.49716	390.903857	-5.924739	9.05E-09	1.27E-06	9.72706284
PRELID2	-16.65945	23.552242	-5.916855	9.45E-09	1.32E-06	9.6865759
PDPK1	12.156492	38.2755656	5.91467984	9.56E-09	1.33E-06	9.67541275
YIPF4	8.50423221	16.264953	5.91438415	9.57E-09	1.33E-06	9.67389553
TMEM56	16.9597283	25.8873437	5.90907953	9.85E-09	1.36E-06	9.64668669

MYL12A	-1428.2128	2215.98789	-5.9067914	9.97E-09	1.37E-06	9.63495614
CDC37	-73.592351	203.416468	-5.904996	1.01E-08	1.37E-06	9.62575397
INO80D	17.0152696	58.2832019	5.90152687	1.03E-08	1.39E-06	9.60797963
AKAP11	19.6032523	67.612341	5.89808215	1.05E-08	1.41E-06	9.59033829
DGKA	10.8800313	28.3342514	5.89764368	1.05E-08	1.41E-06	9.58809337
9-Sep	81.3179753	400.768648	5.89546286	1.06E-08	1.42E-06	9.57692961
TCEAL3	-10.623754	20.9463062	-5.8911976	1.08E-08	1.44E-06	9.55510462
ADAM28	5.34895573	8.07278409	5.8859401	1.12E-08	1.48E-06	9.52821941
GADD45A	-27.012193	47.2057199	-5.8709357	1.21E-08	1.59E-06	9.45159396

GOLIM4	-23.790687	50.4411243	-5.8700305	1.22E-08	1.59E-06	9.44697604
TAX1BP3	-29.452683	44.5761547	-5.8663799	1.24E-08	1.61E-06	9.42835799
DIP2B	16.9856995	53.8741549	5.86571808	1.24E-08	1.61E-06	9.42498385
ARHGEF18	9.94156425	31.4282984	5.86106838	1.28E-08	1.64E-06	9.40128568
PRR12	9.3514538	23.5049469	5.85585957	1.31E-08	1.68E-06	9.37475525
CREBBP	36.387079	158.99549	5.83952335	1.43E-08	1.82E-06	9.29166778
SERF2	-663.87057	915.336144	-5.8383816	1.44E-08	1.82E-06	9.28586752
SERF2-C15ORF63	-663.87057	915.336144	-5.8383816	1.44E-08	1.82E-06	9.28586752

ACTB	-7647.2211	16988.4191	-5.8297055	1.51E-08	1.90E-06	9.24182054
SF3B3	20.7241722	76.555005	5.82684872	1.53E-08	1.92E-06	9.22732805
UBE2G2	14.8882742	55.9341424	5.82615024	1.54E-08	1.92E-06	9.22378556
STK39	-70.965376	147.153418	-5.8240691	1.56E-08	1.93E-06	9.21323261
TPM3	-268.89884	1027.53946	-5.8183262	1.60E-08	1.98E-06	9.18412708
TFEC	12.4838993	30.2318826	5.81228918	1.66E-08	2.03E-06	9.15355465
SERTAD2	12.9560842	47.866563	5.80471067	1.73E-08	2.11E-06	9.11521127
UBE2Q2	-109.4206	198.747289	-5.8017292	1.75E-08	2.13E-06	9.10013738

GPX4	-193.43997	363.601056	-5.7980054	1.79E-08	2.16E-06	9.08131882
BIN2	-294.46284	644.975674	-5.7970247	1.80E-08	2.16E-06	9.07636429
PHF12	12.3052317	48.0312863	5.79051658	1.86E-08	2.23E-06	9.04350164
SERPINB1	-85.370152	198.590708	-5.7821731	1.95E-08	2.32E-06	9.00141401
IKZF1	60.3828175	282.58318	5.77990788	1.97E-08	2.34E-06	8.98999533
SF3B1	41.9879267	222.722982	5.77778426	1.99E-08	2.35E-06	8.97929386
FOXN3	28.077792	136.03584	5.77336925	2.04E-08	2.39E-06	8.95705522
KMT2A	28.3866251	124.312569	5.77303485	2.04E-08	2.39E-06	8.95537139
TIMMDC1	-22.126651	65.2465292	-5.7665015	2.11E-08	2.47E-06	8.92248876

YWHAE	-293.74909	518.168864	-5.7655523	2.12E-08	2.47E-06	8.91771351
POU2AF1	11.8497522	26.2046537	5.74110656	2.42E-08	2.78E-06	8.79495142
MAOB	-6.5680002	8.31690587	-5.7410297	2.42E-08	2.78E-06	8.7945661
VAMP7	-69.927829	124.654272	-5.7355434	2.49E-08	2.85E-06	8.76707165
RAPGEF6	8.24351661	25.4902833	5.73495069	2.50E-08	2.85E-06	8.76410235
FKBP1B	-14.259353	17.2471069	-5.732684	2.53E-08	2.87E-06	8.75274962
STK3	-18.586156	36.1894839	-5.7315685	2.54E-08	2.88E-06	8.74716407
LSMEM1	-10.666142	11.2454955	-5.7301707	2.56E-08	2.88E-06	8.74016615

HIST1H2BJ	-74.50867	101.856563	-5.7280806	2.59E-08	2.90E-06	8.72970489
UIMC1	-166.97699	247.087498	-5.7193153	2.71E-08	3.03E-06	8.68586536
DEK	-77.855071	296.431494	-5.7106104	2.84E-08	3.16E-06	8.64237999
ATP6V1E1	-43.52202	93.1929581	-5.7045172	2.93E-08	3.24E-06	8.61197274
GVINP1	22.9192776	80.6354216	5.7037373	2.95E-08	3.24E-06	8.60808253
PTPRC	80.7585658	163.359208	5.69188331	3.14E-08	3.44E-06	8.54900624
CD27-AS1	-9.6568576	11.4660867	-5.6907492	3.15E-08	3.44E-06	8.54335946
CTNNA1	-31.643821	133.082937	-5.688783	3.19E-08	3.47E-06	8.53357147

ACTG1	-628.04795	2867.42792	-5.6866883	3.22E-08	3.49E-06	8.52314648
KDM2A	21.5452302	105.414791	5.68203551	3.30E-08	3.56E-06	8.50000187
DBP	6.07398201	10.6678853	5.67968832	3.34E-08	3.59E-06	8.48833178
SMAD3	11.7236298	35.5801936	5.67876771	3.36E-08	3.59E-06	8.48375558
ARL13B	-15.315984	32.45979	-5.6743707	3.44E-08	3.65E-06	8.46190674
YLPM1	22.2585696	106.667063	5.67408389	3.44E-08	3.65E-06	8.46048215
MBNL3	144.82608	400.10018	5.67131661	3.49E-08	3.69E-06	8.44673896
RSRC1	-45.709608	106.932486	-5.6702705	3.51E-08	3.69E-06	8.44154498
SKA2	-17.101379	57.5667482	-5.6651687	3.61E-08	3.78E-06	8.41622527

EIF1	-193.7068	489.26299	-5.6619943	3.67E-08	3.83E-06	8.40048019
DPY30	-19.559807	43.5343129	-5.6569002	3.77E-08	3.91E-06	8.3752283
NLRP1	23.5299599	45.033073	5.65506375	3.80E-08	3.92E-06	8.36612916
LOC728392	23.5299599	45.033073	5.65506375	3.80E-08	3.92E-06	8.36612916
PDCD4	44.635694	213.638445	5.65429197	3.82E-08	3.92E-06	8.36230586
EBLN3P	11.7126348	33.6491201	5.65122105	3.88E-08	3.97E-06	8.34709714
C12orf75	-162.59226	234.510821	-5.639852	4.12E-08	4.17E-06	8.29084893
H1F0	-44.755629	86.4713652	-5.6398446	4.12E-08	4.17E-06	8.29081225

FUNDC1	-15.961698	25.0260518	-5.6395844	4.12E-08	4.17E-06	8.28952576
FAM192A	-27.708198	109.264846	-5.6326959	4.27E-08	4.30E-06	8.25549015
ZCCHC2	-22.516223	66.6369046	-5.6233179	4.49E-08	4.50E-06	8.20920694
YWHAQ	-50.019083	153.925614	-5.6190388	4.59E-08	4.59E-06	8.18810859
TOM1L1	-8.2804041	10.2118494	-5.611226	4.78E-08	4.76E-06	8.14961976
ITPRID2	-58.370741	182.914689	-5.608983	4.84E-08	4.79E-06	8.13857781
SERINC5	8.08655049	13.2833383	5.60463235	4.95E-08	4.89E-06	8.1171703
SCRN1	16.2147336	49.8399651	5.59897061	5.09E-08	4.98E-06	8.08933119

MED13L	40.300354	187.222901	5.59872715	5.10E-08	4.98E-06	8.08813458
GIGYF1	11.36543	24.1591107	5.59782459	5.12E-08	4.98E-06	8.08369889
SORL1	41.8325854	72.0613141	5.59782134	5.12E-08	4.98E-06	8.08368291
GLA	-25.006328	31.4244142	-5.5967532	5.15E-08	4.99E-06	8.07843437
IL10RA	27.0628416	42.6663456	5.59610947	5.17E-08	4.99E-06	8.07527131
AKIRIN2	-68.577263	127.711595	-5.58842	5.38E-08	5.18E-06	8.03751283
FAM129C	7.04319335	12.2719108	5.57929201	5.64E-08	5.41E-06	7.99274454
PLEK	-181.22298	519.6519	-5.5708568	5.89E-08	5.63E-06	7.95142546
PHLPP2	6.4578403	14.1852472	5.56748186	6.00E-08	5.70E-06	7.93490794

HIST1H2BH	-66.723089	103.321264	-5.5653334	6.06E-08	5.75E-06	7.92439679
SPEN	31.8407251	137.157024	5.56007031	6.23E-08	5.88E-06	7.89866201
PSD4	18.4738122	63.1828376	5.55866032	6.28E-08	5.90E-06	7.89177085
CALM1	-165.3254	698.920262	-5.5547767	6.40E-08	6.00E-06	7.87279748
RHOH	9.9198571	27.171732	5.55352847	6.45E-08	6.01E-06	7.8667014
LRRFIP2	-72.202794	169.672252	-5.5530076	6.46E-08	6.01E-06	7.86415809
B2M	-6227.4094	8416.29505	-5.5520251	6.50E-08	6.02E-06	7.85936091
RORA	18.2137474	52.2103081	5.5510882	6.53E-08	6.03E-06	7.85478697

TMEM165	-69.749451	105.337249	-5.5493401	6.59E-08	6.06E-06	7.84625475
ETFA	-97.838627	162.674245	-5.5336189	7.14E-08	6.55E-06	7.76961686
ARL4A	18.2321977	39.7793834	5.52930187	7.30E-08	6.67E-06	7.74860235
PCMT1	-29.033004	58.3323101	-5.5266262	7.40E-08	6.74E-06	7.73558446
TTC33	-37.572575	52.5777496	-5.524748	7.47E-08	6.75E-06	7.72644943
SUPT4H1	-19.464171	49.7337926	-5.5239857	7.50E-08	6.75E-06	7.72274247
NDUFA6	-102.73094	162.224667	-5.523779	7.51E-08	6.75E-06	7.72173755

ALDOA	-166.58967	521.937684	-5.5231735	7.54E-08	6.75E-06	7.71879346
ARG2	-13.750503	16.0781569	-5.5230629	7.54E-08	6.75E-06	7.71825545
ZNF592	16.0913385	68.0889357	5.52186822	7.59E-08	6.75E-06	7.71244781
MTRNR2L2	-63.899553	58.3105532	-5.5212438	7.61E-08	6.75E-06	7.7094127
ZFPM2	-53.536393	61.6087186	-5.5210582	7.62E-08	6.75E-06	7.70851065
CELF1	16.1808838	60.9390904	5.51802104	7.74E-08	6.83E-06	7.69375182
PRRC2A	31.8756828	123.793694	5.51195885	7.98E-08	7.02E-06	7.66431279
IGF2R	20.8470814	38.198791	5.51123555	8.01E-08	7.02E-06	7.66080208
TBC1D9	12.371387	39.3622574	5.50685141	8.19E-08	7.16E-06	7.63953035

MSANTD3	-57.67481	86.1411671	-5.5057336	8.24E-08	7.16E-06	7.63410899
H3F3A	-356.88014	731.751536	-5.5056259	8.25E-08	7.16E-06	7.63358662
WDR81	9.60135383	22.7150072	5.50476358	8.28E-08	7.16E-06	7.62940499
RPS7	105.144739	484.86895	5.50405821	8.31E-08	7.17E-06	7.62598487
RNF213	109.437747	319.580416	5.4982638	8.56E-08	7.36E-06	7.59790279
DOCK2	45.4234622	188.01539	5.49533311	8.69E-08	7.44E-06	7.58370849
TMSB4X	-5812.7475	8259.37182	-5.4921853	8.83E-08	7.54E-06	7.5684696
USP24	26.4279795	106.914487	5.48834545	9.01E-08	7.66E-06	7.54988942
LAMTOR1	-123.67941	219.645373	-5.4872114	9.06E-08	7.68E-06	7.54440427

DYNLRB1	-149.63321	226.108303	-5.4858434	9.12E-08	7.71E-06	7.53778817
ARMCX6	-30.207045	46.1392736	-5.4843259	9.20E-08	7.75E-06	7.53045137
BRK1	-63.608095	133.917788	-5.4816427	9.32E-08	7.83E-06	7.5174815
TUBGCP6	6.39127145	7.72400468	5.47818338	9.49E-08	7.94E-06	7.50076841
KHNYN	12.2029827	38.706372	5.47728327	9.53E-08	7.95E-06	7.49642106
CEP70	-39.248694	76.1039211	-5.4739954	9.69E-08	8.06E-06	7.48054606
CLEC2D	14.1136627	40.0327909	5.47201655	9.79E-08	8.12E-06	7.47099507
PER1	14.832015	19.0137392	5.46977203	9.91E-08	8.18E-06	7.46016531

UBE2Q2P1	-15.095558	29.0785773	-5.4614544	1.03E-07	8.51E-06	7.42006375
MAP4K1	12.9227379	37.0140354	5.45342544	1.08E-07	8.84E-06	7.38140072
C7orf55-LUC7L2	-70.679615	148.130685	-5.4523465	1.08E-07	8.86E-06	7.37620863
MAP1LC3B2	-8.7188593	10.5124792	-5.4501901	1.09E-07	8.93E-06	7.36583422
CHD1L	-35.303085	72.057886	-5.4480854	1.11E-07	8.99E-06	7.3557112
DDX17	45.599037	233.419262	5.44757361	1.11E-07	8.99E-06	7.35325034
DBN1	-30.204024	60.5664666	-5.4456241	1.12E-07	9.05E-06	7.34387759
CD22	18.0947066	47.9786802	5.43964579	1.15E-07	9.30E-06	7.31515213

ALDH5A1	9.0299029	18.1852251	5.4382521	1.16E-07	9.34E-06	7.30845916
GNAS	-789.72384	1382.82116	-5.4359963	1.18E-07	9.42E-06	7.29762894
DOCK9	13.3080275	40.4322281	5.43411624	1.19E-07	9.48E-06	7.28860559
DYNC1H1	43.452266	174.056761	5.43182562	1.20E-07	9.56E-06	7.27761505
PRAG1	7.17407919	12.5411702	5.42568998	1.24E-07	9.83E-06	7.24819423
SLTM	-109.34241	323.417874	-5.421562	1.27E-07	1.00E-05	7.2284155
POU2F2	17.2101638	56.8819749	5.41595237	1.30E-07	1.03E-05	7.20155683
CNTLN	-65.109333	111.034465	-5.4140004	1.32E-07	1.03E-05	7.19221606
DRAM2	9.48481995	23.7655161	5.41212493	1.33E-07	1.03E-05	7.18324415

CORO1C	-304.50666	512.838028	-5.4120817	1.33E-07	1.03E-05	7.18303758
MCU	-40.779635	60.4278664	-5.4116044	1.33E-07	1.03E-05	7.18075422
TAGLN2	-1207.6625	1777.17576	-5.4115552	1.33E-07	1.03E-05	7.18051897
SLBP	-20.611064	72.9765607	-5.4104181	1.34E-07	1.04E-05	7.17508104
HIST1H3H	-221.00375	314.921574	-5.4085291	1.35E-07	1.04E-05	7.16604893
SPOCK2	14.3295968	15.5935531	5.40697907	1.36E-07	1.05E-05	7.15863986
RPL10	193.290949	775.240487	5.40693677	1.36E-07	1.05E-05	7.15843768
GSTO1	-31.832553	67.4964479	-5.405419	1.37E-07	1.05E-05	7.15118435

EIF3H	39.3184395	219.34854	5.40500783	1.38E-07	1.05E-05	7.14921962
C16orf72	18.361438	77.512142	5.40194732	1.40E-07	1.06E-05	7.13459945
STRADB	53.4443463	117.367923	5.39751548	1.43E-07	1.08E-05	7.11344029
STAT6	20.5503939	97.8182361	5.39730017	1.43E-07	1.08E-05	7.11241267
SYNRG	14.4675284	62.4251821	5.39719451	1.43E-07	1.08E-05	7.11190841
SETBP1	13.3386395	44.6788171	5.38636485	1.51E-07	1.14E-05	7.06026527
ADAM9	-45.491758	65.7278549	-5.3822729	1.54E-07	1.16E-05	7.04077394
LZIC	-46.929645	88.0751986	-5.3795462	1.57E-07	1.17E-05	7.02779241
RAB8A	-175.44207	431.05141	-5.3756204	1.60E-07	1.19E-05	7.00911124

CYTH1	27.6161494	127.57982	5.37535055	1.60E-07	1.19E-05	7.00782772
FRMD3	-137.09301	176.100337	-5.372282	1.62E-07	1.20E-05	6.99323427
P2RX5	5.03716863	10.4362284	5.37042293	1.64E-07	1.21E-05	6.98439613
MAP1B	-36.254165	61.6535216	-5.3659907	1.68E-07	1.23E-05	6.96333531
BEX4	-8.4100372	17.2742674	-5.3633924	1.70E-07	1.25E-05	6.95099524
UXS1	-142.13579	202.816216	-5.3625119	1.71E-07	1.25E-05	6.94681443
GRK4	-8.5314601	10.7202993	-5.3614297	1.71E-07	1.25E-05	6.94167704
NAPA	-24.985891	77.8247118	-5.3599973	1.73E-07	1.26E-05	6.93487832

GMPR	-56.01256	89.1663506	-5.3593814	1.73E-07	1.26E-05	6.93195513
COMMD7	-22.05322	50.5121303	-5.3548717	1.77E-07	1.28E-05	6.91056188
CCT6P1	-14.027152	18.9973952	-5.3538259	1.78E-07	1.28E-05	6.9056031
GFI1B	-185.47835	268.290987	-5.3538038	1.78E-07	1.28E-05	6.90549799
RUNX2	7.23649375	18.0810184	5.35266649	1.79E-07	1.29E-05	6.90010606
STAU2	-39.176203	74.3938666	-5.3509413	1.81E-07	1.29E-05	6.89192886
VAMP3	-29.609963	89.8971177	-5.3487286	1.83E-07	1.30E-05	6.88144349
ITGAL	29.6477601	97.0569112	5.34581672	1.85E-07	1.32E-05	6.86765063

PDLIM1	-258.60968	412.759091	-5.3457002	1.86E-07	1.32E-05	6.86709879
PTTG1IP	-57.445777	105.987064	-5.3389017	1.92E-07	1.36E-05	6.83492012
SERPINB6	-17.467617	62.5193738	-5.3367335	1.94E-07	1.37E-05	6.82466493
SNRPN	-39.421122	80.6072746	-5.3243991	2.06E-07	1.45E-05	6.766388
RAB6A	-152.80593	382.886008	-5.3242373	2.07E-07	1.45E-05	6.76562399
HIST2H4B	-14.179958	30.704563	-5.3202013	2.11E-07	1.48E-05	6.74657953
PPBP	-5661.4653	6425.09803	-5.3186678	2.12E-07	1.48E-05	6.73934627

OST4	-1233.1338	1438.59432	-5.3146985	2.17E-07	1.51E-05	6.72063189
FAH	-11.413184	16.0946803	-5.3115379	2.20E-07	1.53E-05	6.70573879
DYNLL1	-143.90987	246.451056	-5.3108125	2.21E-07	1.53E-05	6.70232167
VEGFC	-12.210768	19.440433	-5.3105858	2.21E-07	1.53E-05	6.70125374
CDC42	-95.965171	320.591476	-5.3088655	2.23E-07	1.54E-05	6.69315163
CCZ1	-14.886963	58.5497852	-5.3028969	2.30E-07	1.58E-05	6.66505798
11-Sep	-123.06941	204.973318	-5.3020499	2.31E-07	1.58E-05	6.66107292

FAM32A	-27.846269	83.8216901	-5.3011646	2.32E-07	1.58E-05	6.65690873
FGD4	-45.604824	96.5031856	-5.3008321	2.32E-07	1.58E-05	6.6553451
PLA2G4A	-18.794264	29.6357604	-5.2991667	2.34E-07	1.59E-05	6.64751293
MIER1	-66.776413	218.040858	-5.2975799	2.36E-07	1.60E-05	6.64005288
HNRNPH1	38.622172	170.611204	5.29645561	2.37E-07	1.61E-05	6.63476795
CHMP5	-19.799275	70.1572076	-5.289187	2.46E-07	1.66E-05	6.60062351
IQGAP1	54.8415211	294.149283	5.28886685	2.46E-07	1.66E-05	6.59912066
TMEM40	-60.127524	77.1248067	-5.2855485	2.51E-07	1.68E-05	6.58354601

HIST1H2BG	-23.951185	46.5203994	-5.2826651	2.54E-07	1.70E-05	6.57001957
ACTR3B	-21.807082	31.0238985	-5.2810319	2.56E-07	1.71E-05	6.56236044
CAP1	-165.3018	676.500809	-5.2752291	2.64E-07	1.76E-05	6.53516365
PROKR1	-109.17788	169.868846	-5.2737377	2.66E-07	1.76E-05	6.52817787
APLF	-109.17788	169.868846	-5.2737377	2.66E-07	1.76E-05	6.52817787
OXA1L	15.1528719	60.0984741	5.26634583	2.76E-07	1.82E-05	6.49357632
UPF3A	-27.72904	76.0256849	-5.2622003	2.81E-07	1.85E-05	6.47418861

H2AFJ	-37.912325	62.8874893	-5.2544017	2.92E-07	1.92E-05	6.43774953
PDGFA	-78.10604	106.863616	-5.2518537	2.96E-07	1.94E-05	6.42585363
SLC7A6	5.12985931	8.78199647	5.25050294	2.98E-07	1.95E-05	6.4195493
GABARAPL2	-110.34817	257.156919	-5.243422	3.09E-07	2.01E-05	6.38652219
PPP1R14A	-34.720639	49.9949482	-5.2414153	3.12E-07	2.03E-05	6.37716877
ARF1	-189.66762	437.113801	-5.2340005	3.23E-07	2.10E-05	6.34263441
CMPK1	-178.49466	395.732288	-5.2339035	3.24E-07	2.10E-05	6.34218275

HIST2H2BE	-194.94309	297.214666	-5.2334459	3.24E-07	2.10E-05	6.34005308
ARHGDIB	-463.58322	1268.25582	-5.2228013	3.42E-07	2.20E-05	6.29054994
RAB11B	-39.394017	94.2293057	-5.220883	3.45E-07	2.22E-05	6.28163788
BANP	-19.584776	64.6623279	-5.21831	3.49E-07	2.24E-05	6.26968783
HERC3	10.5172015	30.9803878	5.21628506	3.53E-07	2.26E-05	6.26028682
ILF3	20.6110239	93.8879669	5.21283273	3.59E-07	2.29E-05	6.24426578
FANCL	-23.339215	42.8212837	-5.2113925	3.62E-07	2.30E-05	6.23758487
ACER2	-21.339392	27.9935481	-5.2111928	3.62E-07	2.30E-05	6.23665857

PGRMC1	-418.14942	547.096424	-5.2063968	3.71E-07	2.35E-05	6.21442212
PLEKHA8P1	-14.13834	21.9786595	-5.2047037	3.74E-07	2.36E-05	6.20657633
ALDH6A1	5.28941862	8.72708561	5.20437791	3.74E-07	2.36E-05	6.2050668
PTPN12	-400.48853	694.98702	-5.2034294	3.76E-07	2.36E-05	6.20067247
ZHX2	9.26143927	27.879673	5.20283227	3.77E-07	2.37E-05	6.1979064
HCFC1	21.012049	81.2812568	5.20096652	3.81E-07	2.38E-05	6.18926535
PAIP2	-249.32656	494.084147	-5.1998379	3.83E-07	2.39E-05	6.18403949
FMNL1	41.7763747	159.250695	5.19921979	3.84E-07	2.39E-05	6.18117785

EPSTI1	-60.531578	145.004339	-5.197083	3.88E-07	2.41E-05	6.17128721
DIP2A	11.830551	27.502761	5.19671441	3.89E-07	2.41E-05	6.16958169
C9orf16	-32.051268	60.2390785	-5.1916033	3.98E-07	2.46E-05	6.14593921
SNX3	-120.50367	347.73864	-5.1895012	4.03E-07	2.48E-05	6.13622059
UNC13B	-8.8418675	18.0589542	-5.1893262	4.03E-07	2.48E-05	6.13541209
PDLIM7	-21.205558	45.6116719	-5.1878903	4.06E-07	2.49E-05	6.12877549
SBF1	16.0123698	49.8090281	5.18232826	4.17E-07	2.54E-05	6.10308397
SCLT1	-173.1281	321.014931	-5.1819939	4.18E-07	2.54E-05	6.10154024

RALGAPB	13.0127749	49.7656723	5.18126522	4.19E-07	2.54E-05	6.09817625
TPM4	-942.62059	1794.31701	-5.1809766	4.20E-07	2.54E-05	6.09684393
HIST2H4A	-14.417307	31.3092282	-5.1808358	4.20E-07	2.54E-05	6.09619403
ACRBP	-166.39646	220.932214	-5.1806651	4.20E-07	2.54E-05	6.09540594
RHEB	-32.107561	62.1533823	-5.1774244	4.27E-07	2.57E-05	6.08045146
SQSTM1	-49.946583	174.610241	-5.1772253	4.28E-07	2.57E-05	6.07953253
TADA3	-22.148412	87.0319933	-5.1739876	4.34E-07	2.61E-05	6.06459994

VDAC3	-84.909809	145.563083	-5.1722945	4.38E-07	2.63E-05	6.05679413
WASHC4	15.47367	63.1092672	5.16977596	4.43E-07	2.65E-05	6.04518673
CALM3	-653.39197	1097.61679	-5.1688543	4.45E-07	2.66E-05	6.04094011
SEMA7A	4.87431412	9.4616634	5.16529111	4.53E-07	2.70E-05	6.02452849
KLK1	6.93082095	9.1312318	5.16170483	4.61E-07	2.74E-05	6.00801985
AGPAT4	3.12180295	3.30140083	5.15993422	4.65E-07	2.76E-05	5.99987272
ZNF107	9.57951956	25.0953937	5.15944728	4.66E-07	2.76E-05	5.99763259
SNRNP200	34.1027118	173.639049	5.1567047	4.73E-07	2.79E-05	5.98501866
TRAF5	8.85865723	24.9415076	5.151534	4.85E-07	2.85E-05	5.96125214

CDK2AP1	-75.635057	128.19649	-5.1510257	4.86E-07	2.85E-05	5.95891672
SPATA13	18.7845226	69.1849718	5.14958543	4.89E-07	2.87E-05	5.95230082
GRB10	-14.391199	39.2174503	-5.1459721	4.98E-07	2.91E-05	5.93570933
CYTOR	-26.818317	41.318198	-5.1456023	4.99E-07	2.91E-05	5.93401203
PARP8	19.53196	82.8719814	5.14421228	5.02E-07	2.92E-05	5.92763227
PTPN23	6.87713523	18.318054	5.14311124	5.05E-07	2.93E-05	5.92257996
PKD2	-7.8648868	15.2761449	-5.1388876	5.16E-07	2.99E-05	5.9032074
TRANK1	22.2951864	73.5031374	5.13779415	5.18E-07	3.00E-05	5.89819398

FAR2	-9.7996843	21.514966	-5.1291278	5.41E-07	3.12E-05	5.85849188
MAP1LC3B	-150.94882	273.984352	-5.1279104	5.44E-07	3.13E-05	5.85291902
TECPR1	6.7249893	15.0913836	5.12661581	5.47E-07	3.14E-05	5.84699419
STRAP	-21.004866	69.0084106	-5.1261167	5.49E-07	3.14E-05	5.84471049
MTFR1L	-26.108087	45.4804999	-5.1246744	5.52E-07	3.15E-05	5.83811127
LY9	4.99946619	8.70824922	5.12444679	5.53E-07	3.15E-05	5.83707015
MEF2D	13.1992953	56.0696667	5.12085236	5.63E-07	3.20E-05	5.82063199
AP2M1	-83.014537	249.742835	-5.1152423	5.78E-07	3.28E-05	5.79499505

PCMTD2	12.6103861	41.1524608	5.11178964	5.88E-07	3.33E-05	5.77922841
GNG11	-262.86688	370.48222	-5.1111023	5.90E-07	3.34E-05	5.77609093
ZNF106	35.0504336	179.316098	5.10976119	5.94E-07	3.35E-05	5.76996965
PYGL	-61.041742	116.910712	-5.1092361	5.95E-07	3.35E-05	5.7675732
ACOT7	-9.7739704	16.7588988	-5.1082908	5.98E-07	3.36E-05	5.76326008
TAGAP	18.9969137	80.1064083	5.10648908	6.03E-07	3.38E-05	5.7550406
RPS14	55.6947818	264.835544	5.10191856	6.17E-07	3.45E-05	5.73420077
IKBKG	-23.413475	45.4220669	-5.1011257	6.19E-07	3.45E-05	5.73058711

RGS10	-540.32047	675.724842	-5.1010581	6.19E-07	3.45E-05	5.73027893
SH3BGRL	-764.07109	1143.76478	-5.0992431	6.25E-07	3.47E-05	5.72200892
HIST1H2BK	-371.66332	509.659951	-5.0971064	6.31E-07	3.50E-05	5.71227585
OAZ1	-311.36353	803.782424	-5.0943917	6.40E-07	3.54E-05	5.69991504
MED12	11.5193516	42.2175806	5.09395485	6.41E-07	3.54E-05	5.69792618
NEK1	-34.508077	78.9684835	-5.0909206	6.50E-07	3.58E-05	5.68411764
ATP6V0E2	3.25063785	4.54128559	5.09054193	6.52E-07	3.58E-05	5.68239476
DNAJC3	-55.963236	96.5729405	-5.0904361	6.52E-07	3.58E-05	5.68191348

PARD3	-80.436518	116.39026	-5.0897565	6.54E-07	3.58E-05	5.67882143
EEF2	371.023645	1900.05338	5.08738938	6.62E-07	3.61E-05	5.66805583
SVIP	-63.21727	105.078588	-5.0860237	6.66E-07	3.63E-05	5.6618465
C12orf76	-25.644099	37.2237793	-5.0834824	6.74E-07	3.67E-05	5.65029581
CCL5	-1456.6481	2078.75802	-5.0818845	6.80E-07	3.69E-05	5.64303546
ZKSCAN8	8.29525958	22.5287976	5.08035948	6.85E-07	3.71E-05	5.63610787
NOTCH2	31.8495665	35.3063996	5.07814673	6.92E-07	3.74E-05	5.62605943
TMEM91	-25.124337	36.944376	-5.0713181	7.15E-07	3.85E-05	5.59507234

APP	-330.95949	458.583083	-5.0711426	7.16E-07	3.85E-05	5.59427639
ARHGAP10	-107.5431	150.9534	-5.0692662	7.22E-07	3.87E-05	5.5857678
SDCCAG8	-24.895319	70.5892153	-5.0692495	7.22E-07	3.87E-05	5.58569215
FLOT1	-34.567394	120.511276	-5.0671754	7.29E-07	3.90E-05	5.57629032
UFL1	9.29216829	30.4868396	5.06648087	7.32E-07	3.90E-05	5.57314287
ZBTB40	8.87961099	24.4111411	5.06631286	7.32E-07	3.90E-05	5.5723815
MAN1A2	-357.58186	736.923516	-5.0655485	7.35E-07	3.90E-05	5.56891785
DDOST	7.08113938	17.7809409	5.06538933	7.36E-07	3.90E-05	5.56819679

TMED2	-25.425864	83.3485814	-5.0628177	7.45E-07	3.94E-05	5.55654747
NCK1-DT	-5.0619019	6.78050985	-5.0620793	7.48E-07	3.94E-05	5.55320327
AUTS2	7.9392234	23.9546067	5.06187198	7.48E-07	3.94E-05	5.55226467
TTN	25.3023475	25.0475646	5.0570282	7.66E-07	4.03E-05	5.53033944
YY1AP1	-123.06675	360.489244	-5.0561214	7.69E-07	4.04E-05	5.52623686
WDFY2	9.84830135	25.7007521	5.05562436	7.71E-07	4.04E-05	5.5239882
LINC00534	-13.249341	15.2875287	-5.0545934	7.75E-07	4.05E-05	5.51932491
OSBPL2	-11.721078	49.4241098	-5.0522905	7.84E-07	4.09E-05	5.50891098

TMEM140	-70.858864	95.0741157	-5.0514211	7.87E-07	4.10E-05	5.50498091
KIAA0040	13.2840356	47.0318593	5.05041587	7.91E-07	4.11E-05	5.50043698
BRD1	10.3316984	31.0408236	5.04731586	8.02E-07	4.16E-05	5.48642923
GP1BB	-217.45152	288.880706	-5.0471734	8.03E-07	4.16E-05	5.48578572
DOK2	-30.10362	84.2426079	-5.0465907	8.05E-07	4.16E-05	5.4831537
PCYT1B	-53.045521	67.9706804	-5.0461983	8.07E-07	4.16E-05	5.48138097
HLA-DRA	73.409438	270.625756	5.03996965	8.31E-07	4.28E-05	5.45326305
AGPAT1	-80.000829	136.7633	-5.0394231	8.33E-07	4.28E-05	5.45079714

G6PD	-25.180094	78.2126659	-5.0389643	8.35E-07	4.28E-05	5.44872729
PDGFC	-23.148027	32.7497052	-5.0360322	8.47E-07	4.33E-05	5.43550312
PLOD2	-21.695863	34.1015403	-5.0357047	8.48E-07	4.33E-05	5.43402644
ASPH	-37.078904	66.967092	-5.0350634	8.51E-07	4.34E-05	5.43113486
SMIM5	-36.847124	50.4637307	-5.0325244	8.61E-07	4.38E-05	5.41969071
MNT	7.32196058	23.6138974	5.03110114	8.67E-07	4.40E-05	5.41327758
NEXN	-331.63128	419.990065	-5.0290339	8.76E-07	4.44E-05	5.40396523
ZNF460	7.15320318	18.4536705	5.02765083	8.82E-07	4.46E-05	5.39773697

EPS15	-53.598664	243.735626	-5.0269115	8.85E-07	4.46E-05	5.39440794
SNX6	-34.518471	119.583278	-5.0239502	8.98E-07	4.51E-05	5.38107864
RAB11A	-529.67329	796.234494	-5.0236725	8.99E-07	4.51E-05	5.37982903
LOC105370866	-529.67329	796.234494	-5.0236725	8.99E-07	4.51E-05	5.37982903
LGALS12	-16.091253	22.5803254	-5.0215065	9.08E-07	4.55E-05	5.37008405
NUP214	15.1691139	72.5269022	5.01631242	9.31E-07	4.65E-05	5.34672989
CALD1	-115.41309	218.378473	-5.0154267	9.35E-07	4.66E-05	5.34274962
EIF4A2	30.6567454	189.075266	5.01328593	9.44E-07	4.70E-05	5.33313109

MAP3K5	-84.74899	157.265384	-5.0103665	9.58E-07	4.76E-05	5.32001984
KIFAP3	-27.645511	50.0358037	-5.009895	9.60E-07	4.76E-05	5.31790273
LAPTM4B	-9.2410479	12.3789432	-5.0090237	9.64E-07	4.77E-05	5.31399161
GPX1	-360.44758	544.34892	-5.0083319	9.67E-07	4.77E-05	5.31088599
ILK	-103.72692	192.543211	-5.0082358	9.68E-07	4.77E-05	5.31045473
SHOC2	-116.72402	240.288127	-5.0065423	9.75E-07	4.79E-05	5.30285476
MYOM1	-19.761696	28.5068892	-5.0065321	9.75E-07	4.79E-05	5.30280905

LINC00211	-12.470533	16.8280193	-5.0040543	9.87E-07	4.84E-05	5.29169305
TLK2	-17.859695	61.3541879	-5.0032393	9.91E-07	4.85E-05	5.28803784
TARSL2	-13.687358	31.8034834	-5.0011725	1.00E-06	4.89E-05	5.27877092
CNKSR2	2.81436246	3.66501449	4.99795784	1.02E-06	4.95E-05	5.26436309
PDE3B	7.46661558	12.1559998	4.99689611	1.02E-06	4.96E-05	5.25960622
CS	14.7392296	63.4722884	4.99670039	1.02E-06	4.96E-05	5.25872946
SIK1	2.49638936	4.15886381	4.99505546	1.03E-06	4.99E-05	5.2513616
ANO2	-13.576908	39.2778968	-4.9936991	1.04E-06	5.01E-05	5.24528777
SP4	8.83753525	28.5627499	4.99262165	1.04E-06	5.03E-05	5.24046393

RANBP9	-71.412395	171.584191	-4.9909665	1.05E-06	5.06E-05	5.23305554
PTK2	-129.13014	252.386659	-4.9891913	1.06E-06	5.09E-05	5.22511158
NFE2	-52.453074	122.061317	-4.9884418	1.06E-06	5.10E-05	5.22175858
ACTR10	-26.135797	55.7102125	-4.9865192	1.07E-06	5.14E-05	5.21315895
PLCB4	-13.827312	25.2458868	-4.9863248	1.07E-06	5.14E-05	5.21228959
WDR1	-272.49813	642.988917	-4.9819681	1.10E-06	5.23E-05	5.19281375
TEP1	11.5350385	33.9319722	4.98114561	1.10E-06	5.24E-05	5.18913848
FAM53B	16.5666681	63.9618567	4.97872635	1.11E-06	5.30E-05	5.17833118

GNB1	-99.910441	492.618415	-4.9759145	1.13E-06	5.36E-05	5.16577558
ATP6V0E1	-32.297587	66.9013266	-4.9711439	1.15E-06	5.46E-05	5.14448711
LARP1B	-33.658039	64.1273489	-4.971136	1.15E-06	5.46E-05	5.14445218
MSANTD3-TMEFF1	-15.202822	22.3405682	-4.9702144	1.16E-06	5.47E-05	5.14034127
EPC1	9.47066665	30.8905904	4.96306839	1.20E-06	5.65E-05	5.1084899
TRAPPC1	-140.34811	204.051376	-4.9628151	1.20E-06	5.65E-05	5.1073615
AP1M2	-7.5464859	9.20568828	-4.9600789	1.22E-06	5.71E-05	5.09517649

LMNA	-56.201055	106.814829	-4.9576786	1.23E-06	5.77E-05	5.0844918
ATXN2L	19.2091932	75.8133703	4.95594468	1.24E-06	5.80E-05	5.07677591
SH3BGRL3	-461.35582	884.43725	-4.953964	1.25E-06	5.85E-05	5.06796484
CLIC1	-54.998013	238.728519	-4.9507076	1.27E-06	5.92E-05	5.05348537
2-Sep	-105.43654	270.660863	-4.950138	1.27E-06	5.92E-05	5.05095335
SUMO3	-41.027685	122.027713	-4.9500528	1.28E-06	5.92E-05	5.05057477
POLR2E	-16.955974	68.5230902	-4.9499221	1.28E-06	5.92E-05	5.04999364
PSMF1	24.0336895	109.79825	4.9494594	1.28E-06	5.92E-05	5.04793724

HMG20B	-27.447345	67.7607639	-4.9477266	1.29E-06	5.96E-05	5.04023709
LINC01881	-16.157275	26.9140077	-4.9469521	1.29E-06	5.96E-05	5.03679623
SOX6	11.9622028	19.2214777	4.94689596	1.29E-06	5.96E-05	5.03654689
BARD1	-40.457812	77.5664477	-4.9458134	1.30E-06	5.98E-05	5.03173826
ATP5F1A	31.2049344	153.228982	4.9437492	1.31E-06	6.03E-05	5.02257139
CRTC1	4.06492483	7.4208301	4.94122511	1.33E-06	6.09E-05	5.01136666
IVNS1ABP	-39.423706	131.517904	-4.9387319	1.35E-06	6.15E-05	5.00030367
NUTF2	-46.232564	90.4551274	-4.93383	1.38E-06	6.28E-05	4.97856647

NAP1L4	-45.794374	176.96014	-4.9317205	1.39E-06	6.33E-05	4.96921762
CCND3	-90.38547	245.251071	-4.9307732	1.40E-06	6.35E-05	4.96502064
LOC100130357	-5.5480424	6.26497055	-4.927941	1.42E-06	6.43E-05	4.95247598
DAD1	-77.279412	123.511023	-4.9242463	1.44E-06	6.53E-05	4.93612018
PF4	-5445.158	5773.97554	-4.9234286	1.45E-06	6.54E-05	4.93250179
RCSD1	27.495209	155.806375	4.92061006	1.47E-06	6.61E-05	4.92003358
GTF3C6	-26.924658	47.5838162	-4.9205191	1.47E-06	6.61E-05	4.91963124
ATP2B1	12.981109	27.4947326	4.91900874	1.48E-06	6.64E-05	4.91295245

PIP4P2	-52.607964	84.5118818	-4.9179253	1.48E-06	6.66E-05	4.90816234
PIK3R1	23.5619421	133.030224	4.91609078	1.50E-06	6.71E-05	4.90005409
ITFG1	-27.148031	42.2741389	-4.9145941	1.51E-06	6.75E-05	4.89344082
RAB4A	-55.953079	113.309961	-4.9135559	1.51E-06	6.77E-05	4.88885408
PCNP	-74.466434	154.658674	-4.9115497	1.53E-06	6.82E-05	4.87999381
KANSL1	13.5408538	54.130558	4.90663743	1.57E-06	6.97E-05	4.85831112
TPM1	-171.0218	240.702933	-4.9059144	1.57E-06	6.98E-05	4.85512104
PDP1	7.93903563	25.0358221	4.90536654	1.57E-06	6.99E-05	4.8527044

KATNAL1	-16.59203	38.9020743	-4.9047719	1.58E-06	6.99E-05	4.85008169
TMEFF1	-13.466091	18.4470778	-4.9042212	1.58E-06	7.00E-05	4.8476525
CBX6	10.4404311	32.1910144	4.90381158	1.59E-06	7.00E-05	4.84584613
TSC22D1	-578.51522	774.569269	-4.9016573	1.60E-06	7.06E-05	4.83634741
TULP4	19.2959915	70.9616968	4.90051029	1.61E-06	7.09E-05	4.83129161
TMEM158	-13.724082	19.0428518	-4.8990931	1.62E-06	7.12E-05	4.82504615
IPO11	-10.399373	24.2780805	-4.8971953	1.64E-06	7.16E-05	4.81668479
IPO11-LRRC70	-10.399373	24.2780805	-4.8971953	1.64E-06	7.16E-05	4.81668479

ASH2L	-29.941394	100.448949	-4.8928619	1.67E-06	7.29E-05	4.79760362
POLR3G	-51.575918	68.8178125	-4.89208	1.68E-06	7.31E-05	4.79416186
TUBA1C	-71.661156	180.333423	-4.8913178	1.68E-06	7.32E-05	4.79080786
ZMAT2	-69.324445	196.700185	-4.8886724	1.70E-06	7.40E-05	4.77916913
VAPB	-65.225861	141.022036	-4.8878402	1.71E-06	7.41E-05	4.7755088
PARP15	6.80047703	16.425345	4.88733426	1.71E-06	7.41E-05	4.77328397
HIST1H2AC	-688.46698	1075.48064	-4.887205	1.71E-06	7.41E-05	4.77271575

ARMCX3	-33.392157	74.1494451	-4.8870881	1.72E-06	7.41E-05	4.77220153
INKA2-AS1	-14.20965	18.9669877	-4.8821791	1.76E-06	7.57E-05	4.75062408
CCDC50	20.7821933	87.2913094	4.88139748	1.76E-06	7.58E-05	4.74719008
SKAP2	-179.68796	323.482772	-4.8808742	1.77E-06	7.58E-05	4.74489149
TSPAN5	15.5191715	39.726285	4.8803067	1.77E-06	7.58E-05	4.74239869
ZSWIM8	7.32600556	22.1068433	4.8802376	1.77E-06	7.58E-05	4.74209517
CALU	-23.11988	46.3767585	-4.8775982	1.79E-06	7.67E-05	4.7305053
UBAP2	-120.7906	262.02308	-4.874771	1.82E-06	7.75E-05	4.71809616
LINC00926	7.44441286	12.4085272	4.87438556	1.82E-06	7.75E-05	4.71640497

GUCY1B1	-119.10309	172.514393	-4.8741336	1.82E-06	7.75E-05	4.71529943
SPARC	-1157.52	1538.15159	-4.8726983	1.83E-06	7.79E-05	4.70900296
PLCL2	15.6031507	72.9987621	4.87121203	1.85E-06	7.83E-05	4.70248417
LONP2	10.2737224	41.4998353	4.87092694	1.85E-06	7.83E-05	4.70123398
UBXN4	-28.21383	112.831716	-4.8703537	1.85E-06	7.84E-05	4.69872017
ACVR1	-27.703756	41.8538117	-4.8680111	1.88E-06	7.91E-05	4.68845104
AMFR	-45.120915	81.6695426	-4.8655719	1.90E-06	7.98E-05	4.67776238
SLC25A36	8.01648558	22.1044877	4.86542828	1.90E-06	7.98E-05	4.67713314

REPS2	-33.006676	56.7269217	-4.8652052	1.90E-06	7.98E-05	4.67615602
SUSD1	-169.94898	262.687423	-4.8641531	1.91E-06	8.00E-05	4.67154726
NLK	-91.038938	150.278547	-4.8624167	1.92E-06	8.05E-05	4.66394313
PKM	-628.65874	1117.27792	-4.8615858	1.93E-06	8.07E-05	4.66030519
ARL6IP5	-112.85283	184.738379	-4.8614063	1.93E-06	8.07E-05	4.65951914
SEC24C	15.1961676	62.6022936	4.85986837	1.95E-06	8.11E-05	4.65278712
PGD	-85.549007	194.948478	-4.8584888	1.96E-06	8.15E-05	4.64674965
CR1	8.08879179	16.9395809	4.85798455	1.97E-06	8.15E-05	4.64454349

PAPSS1	-17.399182	48.0976804	-4.8575175	1.97E-06	8.15E-05	4.64250027
ITGA9-AS1	-15.953393	22.3474554	-4.8574429	1.97E-06	8.15E-05	4.64217355
QRICH1	13.1508094	62.6350035	4.85693615	1.97E-06	8.15E-05	4.63995679
SKA3	-48.302723	93.6211657	-4.8568372	1.98E-06	8.15E-05	4.63952393
RAB31	-210.91393	340.758314	-4.8522252	2.02E-06	8.31E-05	4.61935718
LIMD1	9.92050174	34.8487961	4.85124819	2.03E-06	8.34E-05	4.61508697
ESAM	-43.080824	62.9327463	-4.8508009	2.03E-06	8.34E-05	4.61313234
TGFB1I1	-14.917416	24.9335059	-4.8483323	2.06E-06	8.42E-05	4.60234717

MAST3	17.7987663	70.3505675	4.84587567	2.08E-06	8.51E-05	4.59161893
TIAM1	10.9747093	37.6187282	4.84377537	2.10E-06	8.58E-05	4.58245043
MEF2C-AS1	-13.649314	20.9795247	-4.8426365	2.11E-06	8.59E-05	4.57748022
LINC00548	-5.6434042	6.78801463	-4.8424639	2.11E-06	8.59E-05	4.57672697
LINC00598	-5.6434042	6.78801463	-4.8424639	2.11E-06	8.59E-05	4.57672697
FCRL1	5.15335217	6.74988569	4.83992781	2.14E-06	8.68E-05	4.56566327
WSB2	-14.107831	51.3512563	-4.8386146	2.15E-06	8.70E-05	4.55993637
SNAP23	-226.07301	372.2468	-4.8385329	2.15E-06	8.70E-05	4.55957994

RANBP10	13.5784762	31.8224399	4.83381544	2.20E-06	8.87E-05	4.53901769
MYL9	-455.38788	598.155443	-4.8337655	2.20E-06	8.87E-05	4.53880009
HIST1H2BC	-148.4824	392.046006	-4.833006	2.21E-06	8.89E-05	4.53549138
ARIH1	13.7030734	60.274105	4.83249812	2.21E-06	8.89E-05	4.53327882
UBE2L3	-25.130257	98.1021824	-4.8322839	2.21E-06	8.89E-05	4.53234584
PWAR5	3.25180694	4.21601	4.83163022	2.22E-06	8.90E-05	4.52949858
PTAR1	8.948338	31.3643178	4.83095546	2.23E-06	8.92E-05	4.52655998
KIF2A	-974.19206	1463.12368	-4.8295992	2.24E-06	8.95E-05	4.52065444
SEZ6L	0.80254284	0.60295509	4.82958552	2.24E-06	8.95E-05	4.52059488

ASAP1	-171.76295	390.030216	-4.8278149	2.26E-06	9.01E-05	4.51288719
ST3GAL3	-20.898527	33.4291112	-4.8253348	2.29E-06	9.10E-05	4.50209509
SGMS1	-24.626309	55.484632	-4.8236556	2.30E-06	9.15E-05	4.49479074
ZNF407	7.70726552	22.3068413	4.82225336	2.32E-06	9.19E-05	4.48869296
RAB2B	18.3546147	44.9602373	4.82207892	2.32E-06	9.19E-05	4.48793449
GRAP2	-510.18145	770.7009	-4.8206614	2.34E-06	9.24E-05	4.481772
LAPTM5	50.4509686	262.923626	4.81912992	2.35E-06	9.29E-05	4.47511542
PSTPIP2	-61.559945	126.802401	-4.8173166	2.37E-06	9.36E-05	4.4672365

PADI4	-24.081819	40.0554176	-4.81644	2.38E-06	9.38E-05	4.46342821
SLC25A3	23.6579931	107.542206	4.81478415	2.40E-06	9.44E-05	4.45623679
BCL2	15.7798959	57.9638286	4.81444448	2.41E-06	9.44E-05	4.45476187
SMARCA5	-224.84141	519.852569	-4.8136642	2.41E-06	9.46E-05	4.45137377
CTSA	-322.90034	457.160207	-4.8126059	2.43E-06	9.49E-05	4.44677967
CGRRF1	-8.8970595	15.3418369	-4.8095802	2.46E-06	9.61E-05	4.43364929
CD63	-20.961808	44.1993648	-4.8091789	2.46E-06	9.61E-05	4.43190819
CHMP4B	-36.92258	150.205775	-4.8072936	2.49E-06	9.68E-05	4.42373065

SBDS	-29.477019	75.5358597	-4.8054692	2.51E-06	9.74E-05	4.41582007
DNAJC4	-15.952133	37.0440982	-4.8053084	2.51E-06	9.74E-05	4.41512292
TTC14	6.70902756	6.95209518	4.80327476	2.53E-06	9.82E-05	4.40630799
RHOBTB1	-81.996676	118.047699	-4.8014412	2.55E-06	9.89E-05	4.39836302
